# Tetracycline-induced mitohormesis mediates disease tolerance against influenza

**DOI:** 10.1172/JCI151540

**Published:** 2022-09-01

**Authors:** Adrienne Mottis, Terytty Y. Li, Gaby El Alam, Alexis Rapin, Elena Katsyuba, David Liaskos, Davide D’Amico, Nicola L. Harris, Mark C. Grier, Laurent Mouchiroud, Mark L. Nelson, Johan Auwerx

**Affiliations:** 1 Laboratory of Integrative Systems Physiology, Institute of Bioengineering, École Polytechnique Fédérale de Lausanne, Lausanne, Switzerland.; 2 Nagi Bioscience SA - EPFL Innovation Park, Écublens, Switzerland.; 3 Laboratory of Intestinal Immunology, Global Health Institute, École Polytechnique Fédérale de Lausanne, Lausanne, Switzerland.; 4 Department of Immunology and Pathology, Central Clinical School, Monash University, Melbourne, Victoria, Australia.; 5 Echelon Biosciences, Inc., Salt Lake City, Utah, USA.

**Keywords:** Infectious disease, Drug therapy, Influenza, Mitochondria

## Abstract

Mitohormesis defines the increase in fitness mediated by adaptive responses to mild mitochondrial stress. Tetracyclines inhibit not only bacterial but also mitochondrial translation, thus imposing a low level of mitochondrial stress on eukaryotic cells. We demonstrate in cell and germ-free mouse models that tetracyclines induce a mild adaptive mitochondrial stress response (MSR), involving both the ATF4-mediated integrative stress response and type I interferon (IFN) signaling. To overcome the interferences of tetracyclines with the host microbiome, we identify tetracycline derivatives that have minimal antimicrobial activity, yet retain full capacity to induce the MSR, such as the lead compound, 9-*tert*-butyl doxycycline (9-TB). The MSR induced by doxycycline (Dox) and 9-TB improves survival and disease tolerance against lethal influenza virus (IFV) infection when given preventively. 9-TB, unlike Dox, did not affect the gut microbiome and also showed encouraging results against IFV when given in a therapeutic setting. Tolerance to IFV infection is associated with the induction of genes involved in lung epithelial cell and cilia function, and with downregulation of inflammatory and immune gene sets in lungs, liver, and kidneys. Mitohormesis induced by non-antimicrobial tetracyclines and the ensuing IFN response may dampen excessive inflammation and tissue damage during viral infections, opening innovative therapeutic avenues.

## Introduction

Cells constantly monitor the function of their mitochondria and activate adaptive mitochondrial stress responses (MSRs) to maintain or restore mitochondrial homeostasis upon stress. Mitohormesis is the phenomenon that ensues when these adaptive responses surpass the initial stress and lead to overall beneficial consequences for cellular and organismal fitness. A prototypical and well-studied form of the MSR is the mitochondrial unfolded protein response (UPR^mt^), first described in mammalian cells (1), but more extensively characterized in *Caenorhabditis elegans* (reviewed in refs. 2–4). Fitting with a beneficial health impact, the mitohormetic induction of the MSR is reported to improve health and extend lifespan in *C*. *elegans* (5, 6), as well as to attenuate the phenotypic consequences of Alzheimer’s disease and exert cardioprotective effects in mouse models (7, 8). Interestingly, tetracyclines (Tets) — antibiotics that not only block bacterial, but also mitochondrial translation — can be used to induce such a mild proteotoxic mitochondrial stress. Tets are therefore pharmacological tools that induce the MSR (7, 9), often resulting in a beneficial mitohormetic response.

Mitochondrial function and immunity, both innate and adaptive, are interconnected at multiple levels (10, 11). Mitochondrial metabolism is a central determinant of the type and course of immune response, and damaged mitochondria contribute to inflammation through the release of damage-associated molecular patterns (DAMPs), among other mechanisms (12). In addition, mitochondria can be targeted by multiple bacterial as well as viral infections (13). Mitochondrial function has been proposed to be essential to trigger tolerance to infection (14, 15). Resistant hosts fight infection by eliciting an immune response that reduces pathogen load, whereas tolerance refers to the mechanisms that limit the extent of organ dysfunction and tissue damage caused by infection, not necessarily affecting pathogen load (16).

Respiratory viruses such as influenza A virus (IFV) or SARS-CoV-2 represent a major public health concern, as our aging population is highly susceptible to the complications and often lethal consequences of such infections (17). Uncontrolled systemic inflammation ensuing from infection by respiratory viruses can lead to acute respiratory distress syndrome (ARDS) and multiorgan dysfunction syndrome (MODS), which are both in part driven by mitochondrial dysfunction (18, 19), contributing significantly to complications and mortality.

We here explore the potential of Tets to induce mitohormesis and disease tolerance within the context of respiratory infection caused by IFV. To dissociate the impact of Tets on the microbiome from potential effects on mitohormesis and tolerance, we profiled the transcriptomic response to doxycycline (Dox) in germ-free mice and show that the Tet-induced MSR crosstalks with the innate immune system, in particular with type I interferon (IFN) signaling. We then assess and select Tet derivatives, devoid of antibacterial activity, for their ability to trigger the MSR in worms and cells. We finally provide proof of concept that a non-antibacterial Tet, substituted at the C9 position, named 9-*tert*-butyl doxycycline (9-TB), induces disease tolerance and increases the survival of mice infected with a lethal dose of IFV by lowering systemic and local inflammation, and limiting lung tissue damage, without affecting the gut microbiome.

## Results

**Figure 1:**
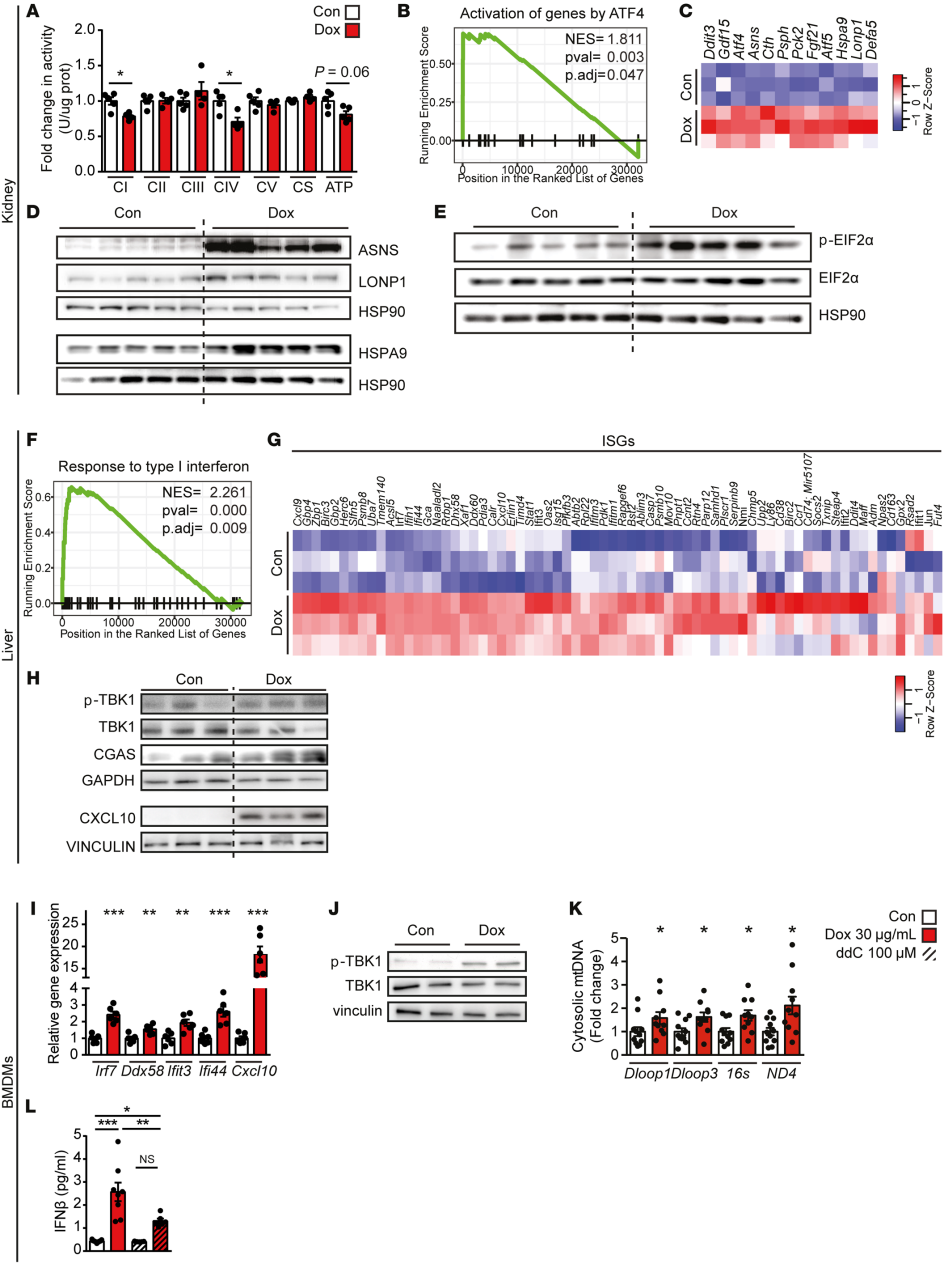
Doxycycline induces the ATF4 response and the type I IFN response. (**A**) Biochemical measurement of oxidative phosphorylation (OXPHOS) complexes (CI–CV), citrate synthase (CS), and ATP levels in the kidney of germ-free C57BL/6J male mice raised and maintained in a germ-free environment and that were drinking regular water or water supplemented with doxycycline (Dox) at 500 mg/kg/day (mpkd) for 16 days (*n =* 4–5). (**B** and **C**) Enrichment score plot for the gene set “Reactome Activation of genes by ATF4” (**B**) and heatmap representing the transcript levels of ATF4/5 targets (**C**) from kidney transcriptomics data of control versus Dox-treated germ-free mice. (**D**) Western blot analysis of selected ATF4 targets in the kidneys of germ-free mice (corresponding loading control below, HSP90). (**E**) Immunoblots of phosphorylated EIF2α (p-EIF2α) and total EIF2α in kidneys of Dox-treated germ-free mice. (**F** and **G**) Enrichment score plot for the GO term “Response to type I interferon” (**F**) and heatmap representing the transcript levels of some IFN-stimulated genes (ISGs) (**G**) from livers of germ-free mice treated with Dox. (**H**) Immunoblots of phosphorylated TBK1 (p-TBK1), TBK1, and the ISG proteins CGAS and CXCL10 (corresponding loading control below, vinculin and GAPDH, respectively). (**I**) Transcript levels of selected ISGs of bone marrow–derived macrophages (BMDMs) (day 6 of differentiation, derived from C57BL/6J mice) treated with Dox at 30 μg/mL for 9 hours (*n =* 6). (**J**) Immunoblots of phosphorylated TBK1 (p-TBK1), TBK1, and vinculin as control in BMDMs treated with Dox at 30 μg/mL for 3 hours. (**K**) Amplification of different mtDNA regions by qPCR in the cytosolic fraction of BMDMs with Dox at 30 μg/mL for 1 hour (*n =* 10). (**L**) Levels of IFN-β in the culture medium of BMDMs treated with Dox (30 μg/mL for 14 hours) and/or 2′,3′-dideoxycytidine (ddC, at 100 μM for 72 hours) (*n =* 8). Statistical analysis: Wilcoxon’s test *P* values corrected for multiple comparisons with Hommel’s method (**A**, **I**, and **K**) or by 1-way ANOVA followed by Tukey’s post hoc correction (**L**). *P ≤ 0.05; **P ≤ 0.01; ***P ≤ 0.001. NS, *P* > 0.05. Error bars represent ±SEM.

To characterize the MSR induced by Tets in vivo, we administered Dox at 500 mg/kg/day (mpkd) in the drinking water to 9-week-old germ-free C57BL/6J mice for 16 days (5, 7), hence eliminating the potential confounding impacts of Dox on the microbiome. Body weight at the time of the sacrifice was not different between the control and Dox-treated animals (Supplemental Figure 1A; supplemental material available online with this article; https://doi.org/10.1172/JCI151540DS1), indicating the absence of obvious adverse effects. As reported in livers of mice maintained under conventional conditions (9), oxidative phosphorylation (OXPHOS) complex activity as well as ATP levels were also reduced by Dox in kidneys of germ-free mice (Figure 1A). Dox elicited organ-specific transcriptional responses, with the expression of quantitatively more and qualitatively different genes being affected in the kidney compared with the liver (Supplemental Figure 1, B and C, and Supplemental Table 1). In Dox-treated kidneys, gene set enrichment analysis (GSEA) (20) revealed the induction of the ATF4-mediated integrated stress response (ISR) (Figure 1B), a common hallmark of the mammalian MSR (21). The MSR features the induction of mitochondrial chaperones and proteases, such as HSPA9 and LONP1, as well as of enzymes mediating adaptation to nutrient deprivation, such as asparagine synthetase (ASNS), which were increased at both the transcript and protein levels (Figure 1, C and D, and Supplemental Figure 1D). In line with the activation of the ATF4/ISR pathway, the kidney displayed increased eIF2α phosphorylation (Figure 1E and Supplemental Figure 1E), which slows down cytosolic cap-dependent translation as a compensation for energy deprivation caused by mitochondrial stress and favors the translation of ATF4 transcripts by cap-independent mechanisms (22). Kidneys of the Dox-treated germ-free mice thus displayed the typical attributes of the ATF4/ISR pathway, a hallmark of the mammalian response to mitochondrial stress.

In the liver, eIF2α phosphorylation and the ATF4/ISR program were not induced (Supplemental Figure 1, F and G, and Supplemental Table 2). The liver transcriptome, however, indicated that Dox induced the type I IFN response (Figure 1, F and G), which was confirmed at the protein level by the increased expression of 2 IFN-stimulated genes (ISGs), cyclic AMP-GMP synthetase (CGAS) and C-X-C motif chemokine ligand 10 (CXCL10), and the increased phosphorylation of TANK-binding kinase 1 (TBK1) (Figure 1H and Supplemental Figure 1H). The type I IFN response is an innate immune pathway that, upon sensing viral DNAs, activates cGAS/STING/TBK1 signaling, culminating in the secretion of the type I IFNs, IFN-α and IFN-β, and the induction of the expression of ISGs (23). Of note, the type I IFN response was also induced in kidneys, but to a lesser extent (Supplemental Figure 1, I and J, and Supplemental Table 2).

Similarly, in mouse bone marrow–derived macrophages (BMDMs), a highly relevant model to investigate innate immune signaling in vitro, Dox induced the expression of ISGs and triggered the phosphorylation of TBK1 (Figure 1, I and J, and Supplemental Figure 1K). Mitochondrial DNA (mtDNA) effusing from the mitochondria into the cytosol was previously shown to elicit the type I IFN response in the context of mitonuclear genomic instability caused by the loss of function of transcription factor A mitochondrial (TFAM) (12). We also detected increased levels of mtDNA in the cytosol of Dox-treated BMDMs (Figure 1K), which underpins the activation of antiviral signaling resulting in the secretion of IFN-β from these BMDMs (Figure 1L). Finally, Dox-induced secretion of IFN-β was abrogated by the nucleoside analogue, 2′,3′-dideoxycytidine (ddC) (Figure 1L), which gradually leads to depletion of mtDNA (24), demonstrating that cytosolic release of mtDNA contributes to the activation of antiviral signaling.

**Figure 2:**
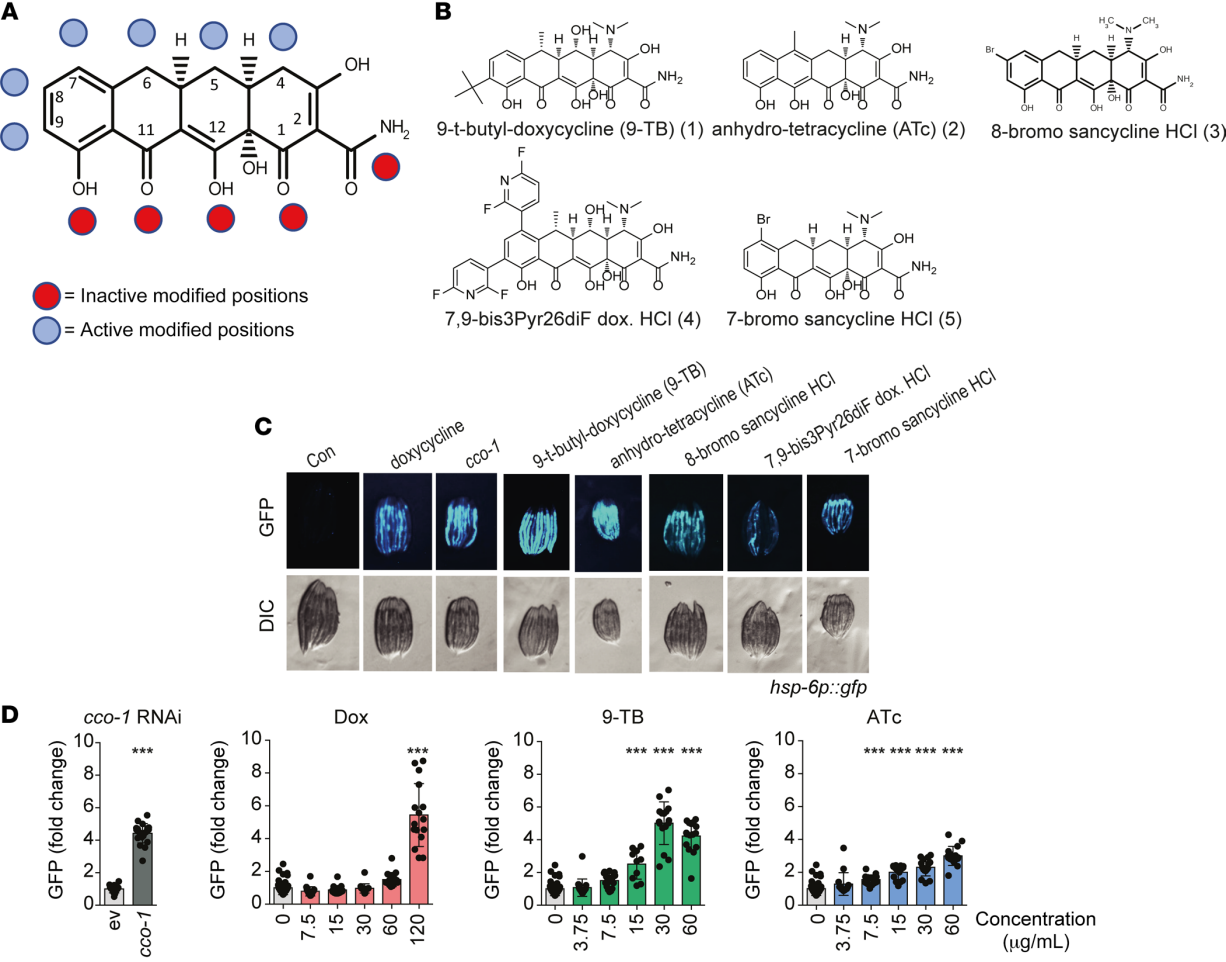
Selecting Tet derivatives that induce UPR^mt^ in *C*. ***elegans***. (**A**) Structural locants of the Tet scaffold and UPR^mt^-active and -inactive compounds by chemically modified positions (based on activity of the *hsp-6:gfp* reporter, **C**). (**B**) Chemical structures of the Tet derivatives shown in **A** and **C**. (**C**) Representative images of the induction of the UPR^mt^ in the *C*. *elegans hsp-6:gfp* reporter strain (25) exposed to the indicated Tet derivatives at 68 μM (except for 9-TB, which is at 17 μM) since the parental L4 stage. Dox and OXPHOS loss of function through feeding *cco-1* RNAi serve as positive controls. The pictures show the progeny at day 2–3 of adulthood (similar exposure time for all images; GFP fluorescence in top raw, differential interference contrast [DIC] in bottom raw). (**D**) Dose-response for the UPR^mt^ activation (*hsp-6:gfp* reporter strain) upon exposure to different concentrations of Dox, 9-TB, ATc, or treatment with *cco-1* RNAi using an automated microfluidic device (33) (*n =* 14–16). Statistical analysis was performed by 1-way ANOVA followed by Bonferroni’s post hoc correction. ***P ≤ 0.001. Error bars represent ±standard deviation (±SD).

We then set out to identify Tets with lower antimicrobial activities that could be more easily developed for clinical use. To this end, we screened in *C*. *elegans* a library of 52 position-modified Tet derivatives (Supplemental Table 3) that are clinically used, are synthetic intermediates, or derivatives specifically synthesized to probe initial structure-activity relationships among Tets that elicit the MSR (Figure 2, A–C), most of them having very limited antibacterial activity (Table 1). We screened the compounds for induction of the UPR^mt^ using a *C*. *elegans* *hsp-6:gfp* reporter strain with Dox administration and *cco-1* RNAi feeding as positive controls, as previously described (25) (Figure 2C). Out of the 52 Tet derivatives tested (Supplemental Table 3), representing clinically relevant and C2–C10 position–modified compounds (26–32) (Figure 2, A and B), we identified 9-TB and anhydrotetracycline 2 (ATc) as the strongest activators of the UPR^mt^ (Figure 2C). We then compared detailed dose responses of Dox, 9-TB, and ATc to induce a GFP signal in the *C*. *elegans* *hsp-6:gfp* reporter strain in an automated microfluidic device (33). 9-TB and ATc were again in this system more efficacious at lower doses to induce the UPR^mt^ relative to Dox (Figure 2D and Supplemental Figure 2A), with 9-TB surpassing the robust UPR^mt^ activation caused by *cco-1* RNAi feeding (Figure 2, C and D).

In addition, other derivatives modified along the upper periphery spanning positions C2, C4, C5, C6, C13, and aromatic positions C7–C9 also activated the UPR^mt^, such as compounds 3–5, although their effect was not as pronounced as that of 9-TB and ATc (Figure 2, A–C). In contrast, clinically used minocycline 7, Nuzyra 14 (see Supplemental Table 3), Tygacil 23, or derivatives based on the minocycline scaffold did not activate the UPR^mt^ (16 compounds), while C5–C9 derivatives of sancycline only mildly activated the UPR^mt^ (see Supplemental Table 3). Additionally, compounds modified at the lower periphery, spanning positions C10, C11, C12-C1, and the A-ring C2 carboxamide did not induce the activity of the GFP reporter, showing the importance of this integrated phenolic keto-enol system in maintaining UPR^mt^ activity (34, 35).

**Figure 3:**
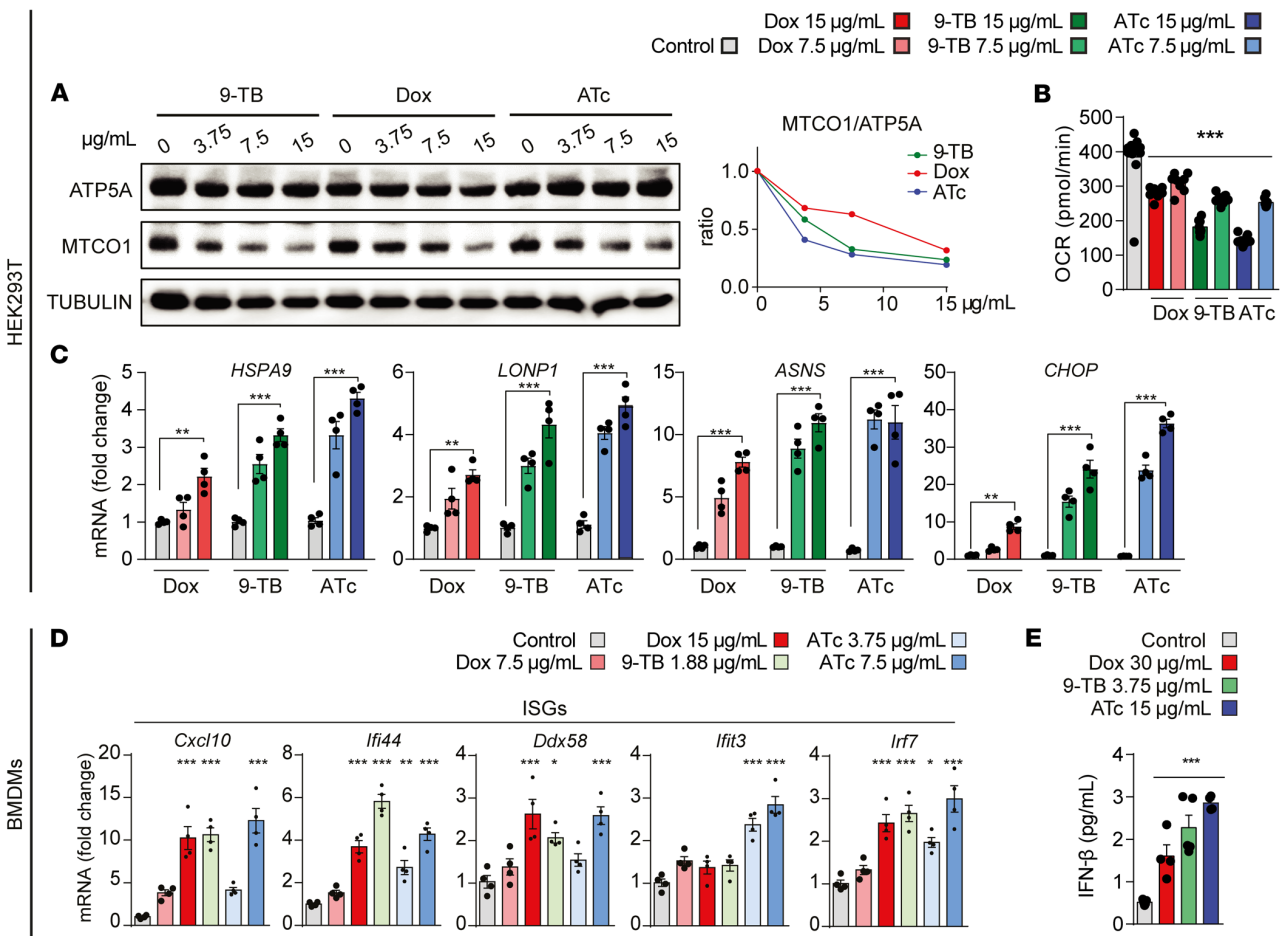
Tet derivatives induce the MSR and type I IFN signalling in mammalian cells. (**A**–**C**) Tet derivatives induce a mitochondrial/nuclear protein imbalance and the MSR in HEK293T cells (human) treated for 24 hours at the indicated concentrations. (**A**) Immunoblots of HEK293T cells for the OXPHOS subunits ATP5A (encoded in nuclear DNA) and MTCO1 (encoded in mtDNA) with tubulin serving as a control. Quantification of the relative MTCO1/ATP5A ratio is shown on the right. (**B**) Oxygen consumption rate of HEK293T cells exposed to different concentrations of Dox, 9-TB, or ATc (*n =* 8). (**C**) Transcript levels of the indicated MSR genes measured by RT-qPCR (*n =* 4). (**D** and **E**) Tet derivatives induce transcript levels of the indicated ISGs (**D**) and stimulate IFN-β secretion (**E**) after 24 hours of treatment at the indicated concentrations in mouse BMDMs (day 6 differentiation) (*n =* 4). Statistical analysis was performed by 1-way ANOVA (**B**, **D**, and **E**) or 2-way ANOVA (**C**) followed by Tukey’s post hoc test . *P ≤ 0.05; **P ≤ 0.01; ***P ≤ 0.001. Error bars represent ±SEM.

We then characterized the pharmacology of Dox, 9-TB, and ATc in the human embryonic kidney (HEK293T) cell line. 9-TB and ATc also generated a more robust MSR response than Dox, as reflected by their impact (up to almost 2-fold stronger) on the mitonuclear protein imbalance (Figure 3A), an imbalanced ratio between mitochondrial and nuclear encoded OXPHOS subunits, underpinning the induction of the MSR (5). Furthermore, 9-TB and ATc reduced the basal oxygen consumption rate (OCR) in a dose-dependent and more pronounced fashion than Dox (Figure 3B). The induction of transcripts for the mammalian MSR signature genes was likewise more prominent with 9-TB and ATc (Figure 3C). In mouse BMDMs, lower doses of 9-TB (1.88 μg/mL) and ATc (3.75 and 7.5 μg/mL) were also superior to Dox (at 7.5 and 15 μg/mL) in inducing the ISG and MSR genes (Figure 3D and Supplemental Figure 3A) and the secretion of IFN-β (Figure 3E). Knocking out ATF4 in mouse embryonic fibroblasts (MEFs) showed that Tets induced the MSR and most ISG genes in an ATF4-dependent manner (Supplemental Figure 3B). Taken together, these studies in *C*. *elegans*, mouse BMDMs, human HEK293T cells, and MEFs ascertained the identification of non-antimicrobial Tets with higher potency to trigger the MSR and type I IFN response, relative to our benchmark antibacterial Tet, Dox.

**Figure 4:**
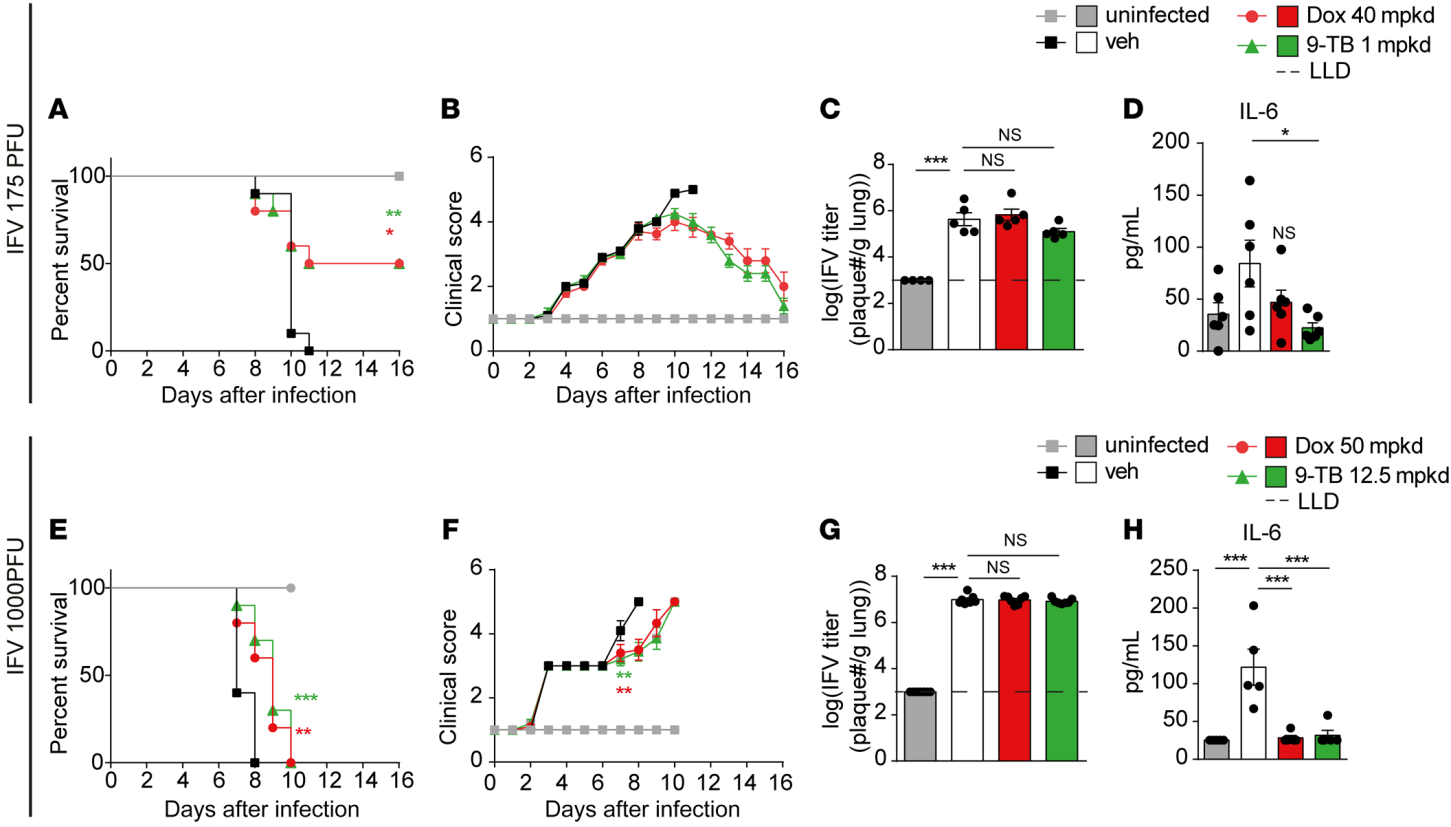
Tets mediate disease tolerance to IFV in mice. (**A**–**D**) Eight-week-old BALB/cN mice were injected with Dox (40 mpkd) or 9-TB (1 mpkd) and intranasally infected with 175 PFU of IFV H1N1 PR8, as described in Supplemental Figure 4A. Survival (**A**) and clinical score (**B**) were followed for 16 days after infection (*n =* 10). On day 7 after infection, viral titers in lung lysates (**C**, *n =* 5) and IL-6 levels in plasma (**D**, *n =* 6) were measured (*n =* 5). (**E**–**H**) Eight-week-old BALB/cN mice were injected with Dox (50 mpkd) or 9-TB (12.5 mpkd) and intranasally infected with 1000 PFU of IFV H1N1 PR8, as shown in Supplemental Figure 4B. Survival (**E**) and clinical score (**F**) were followed over 10 days after infection (*n =* 10). On day 5 after infection, viral titers in lung lysates (**G**) and IL-6 levels in plasma (**H**) were measured (*n =* 5). Dashed horizontal lines in **C** and **G** indicate the lower limit of detection (LLD). Statistical analysis was performed by 1-way ANOVA followed by Tukey’s post hoc test. For survival curves in **A** and **E**, statistical analysis was performed by log-rank (Mantel-Cox) test. *P ≤ 0.05; **P ≤ 0.01; ***P ≤ 0.001. NS, *P* > 0.05. Error bars represent ±SEM.

mtDNA instability–driven innate immunity can potentiate resistance to viruses (12) and mediates the antiviral immune response against the IFV (36). We thus asked whether the Tet-induced MSR enables mice to survive infection by IFV. We hence subjected 8-week-old female BALB/cN mice to either mock (1 group, *n =* 10) or intranasal inoculation with 175 PFU of the IFV H1N1 PR8 strain (3 groups). The 3 infected groups (*n =* 10 each) were given vehicle, Dox (at 40 mpkd), or 9-TB (at 1 mpkd) by daily intraperitoneal injection, from preinoculation day –3 (Supplemental Figure 4A). Dox and 9-TB improved the survival to the infectious challenge, with 50% of the mice treated with Dox or 9-TB recovering (Figure 4A). The improved health of the Tet-treated cohorts was further supported by the recovery of body weight loss, and their improved clinical score (Figure 4B and Supplemental Figure 4C). In contrast, the IFV infection was lethal to all control mice by day 11 after inoculation. Strikingly, on day 7 after infection no significant difference in viral titer in the lung tissue was observed (Figure 4C). Similarly, when mice were infected with a much higher viral load (1000 PFU; Supplemental Figure 3B), Dox and 9-TB delayed mortality and the decline in health (Figure 4, E and F, and Supplemental Figure 4E), again in the absence of an impact on the viral titer in the lungs on day 5 after infection (Figure 4G). The Tet-induced MSR did not cause obvious adverse effects (Supplemental Figure 4D), yet decreased the levels of interleukin 6 (IL-6) in both 175 and 1000 PFU experiments (Figure 4, D and H), and of some other markers of tissue stress and damage (Supplemental Figure 4F) (37–39). These results demonstrate that the Tet-induced MSR increases the survival of mice to a lethal IFV infection by improving tolerance, rather than by reducing viral load, which is reflective of resistance to the virus.

**Figure 5:**
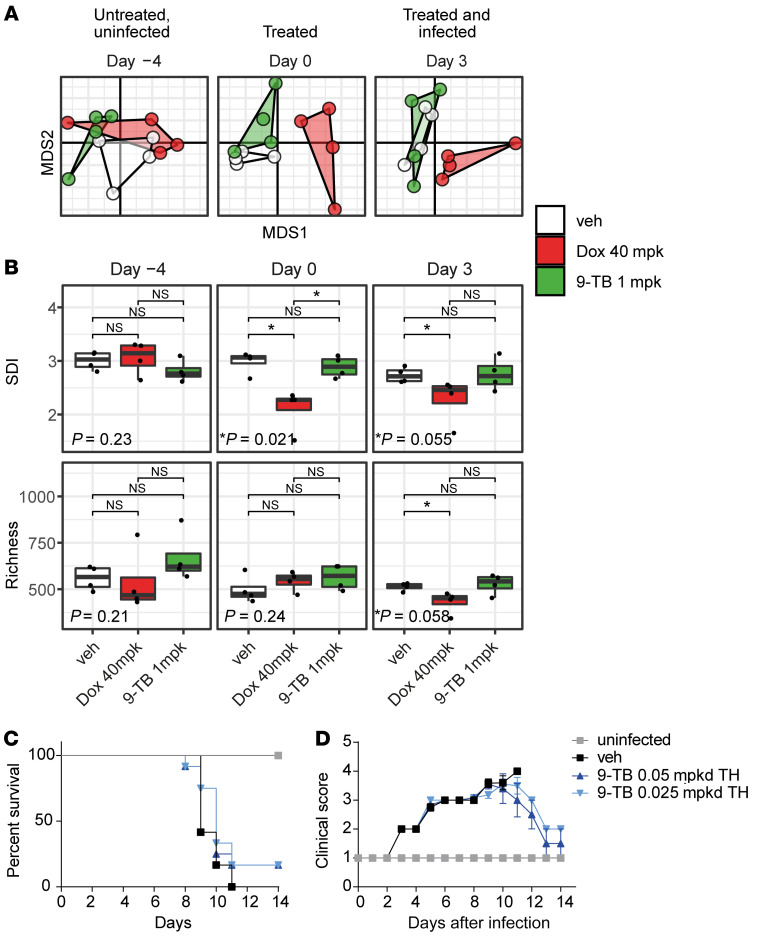
9-TB does not impact gut microbiome and shows encouraging effects when therapeutically administered. (**A**) Comparison of bacterial community composition by nonmetric multidimensional scaling (NMDS) based on the Bray-Curtis dissimilarity. (**B**) Comparison of bacterial species diversity in terms of Shannon diversity index (SDI) and richness. The lower and upper hinges are the first and third quartiles. The middle line is the median. The upper and lower whiskers respectively represent the highest and lowest values that are within 1.5× IQR from the hinge, where IQR is the interquartile range (i.e., distance between the first and third quartile). Data points beyond whiskers are considered outliers. Statistical significance assessed by Kruskal-Wallis test and post hoc Wilcoxon’s test with *P* values adjusted for multiple comparison using the Holm-Bonferroni method. *P < 0.05, **P < 0.01, ***P < 0.001, ****P < 0.0001. NS, *P* > 0.05. (**C** and **D**) Eight-week-old BALB/cN mice (*n =* 12) were infected intranasally with 760 PFU of IFV H1N1 PR8 and injected with 9-TB (0.05, 0.025 mpkd), as described in Supplemental Figure 5A. Survival (**C**) and clinical score (**D**) were followed for 14 days after infection (*n =* 12). Statistical analysis was performed by log-rank (Mantel-Cox) test (**C**) or 1-way ANOVA followed by Tukey’s post hoc test (**D**). Error bars represent ±SEM.

To assess the impact of Tets on the microbiome we transiently individually caged animals and longitudinally collected feces before (day –4, before IFV inoculation), 3 days after (day 0, just before IFV inoculation), and 6 days after (day 3, after IFV inoculation) the start of daily administration of 9-TB or Dox. We then extracted DNA from feces and performed whole-metagenome sequencing. While the composition and diversity of the bacterial communities showed no differences between groups before treatment, the gut bacterial community of mice treated with Dox showed a significant difference in composition compared with both untreated mice and mice treated with 9-TB after 3 and 6 days of Tet treatment (respectively day 0 and day 3 after inoculation), as assessed by permutational multivariate analysis of variance (perMANOVA) and visualized by nonmetric multidimensional scaling (NMDS) (Figure 5A and Supplemental Table 8). This was reflected by a lower bacterial species diversity in Dox-treated mice in terms of both Shannon diversity index (SDI) and richness (Figure 5B). In contrast, no differences were observed between 9-TB–treated mice and untreated mice at any time point, suggesting that that the administered dose of 9-TB does not affect the mice gut microbiota in vivo (Figure 5, A and B, and Supplemental Table 8).

To further investigate the clinical relevance of the Tet derivatives we focused on 9-TB and administered 9-TB in a therapeutic mode starting on day 1 after inoculation with 760 PFU of the IFV H1N1 PR8 strain (Supplemental Figure 5A). Of note, the different death kinetics and survival proportion with regard to the high viral load (760 PFU) in Figure 5, C and D and Supplemental Figure 5, A–D in comparison with Figure 4, A and E at lower viral load (175 PFU) are due to the fact that both experiments were run with different viral batches. Nevertheless, although not significant, the trend of 20% survival upon administration of 2 very low doses of 9-TB (0.025 and 0.05 mpkd) are highly encouraging (Figure 4, C and D) and suggest that these Tet derivatives can trigger tolerance to IFV in a clinically relevant setting. Future investigations are thus needed to optimize the timing and doses of Tet derivatives and refine their therapeutic potential in viral infections.

**Figure 6:**
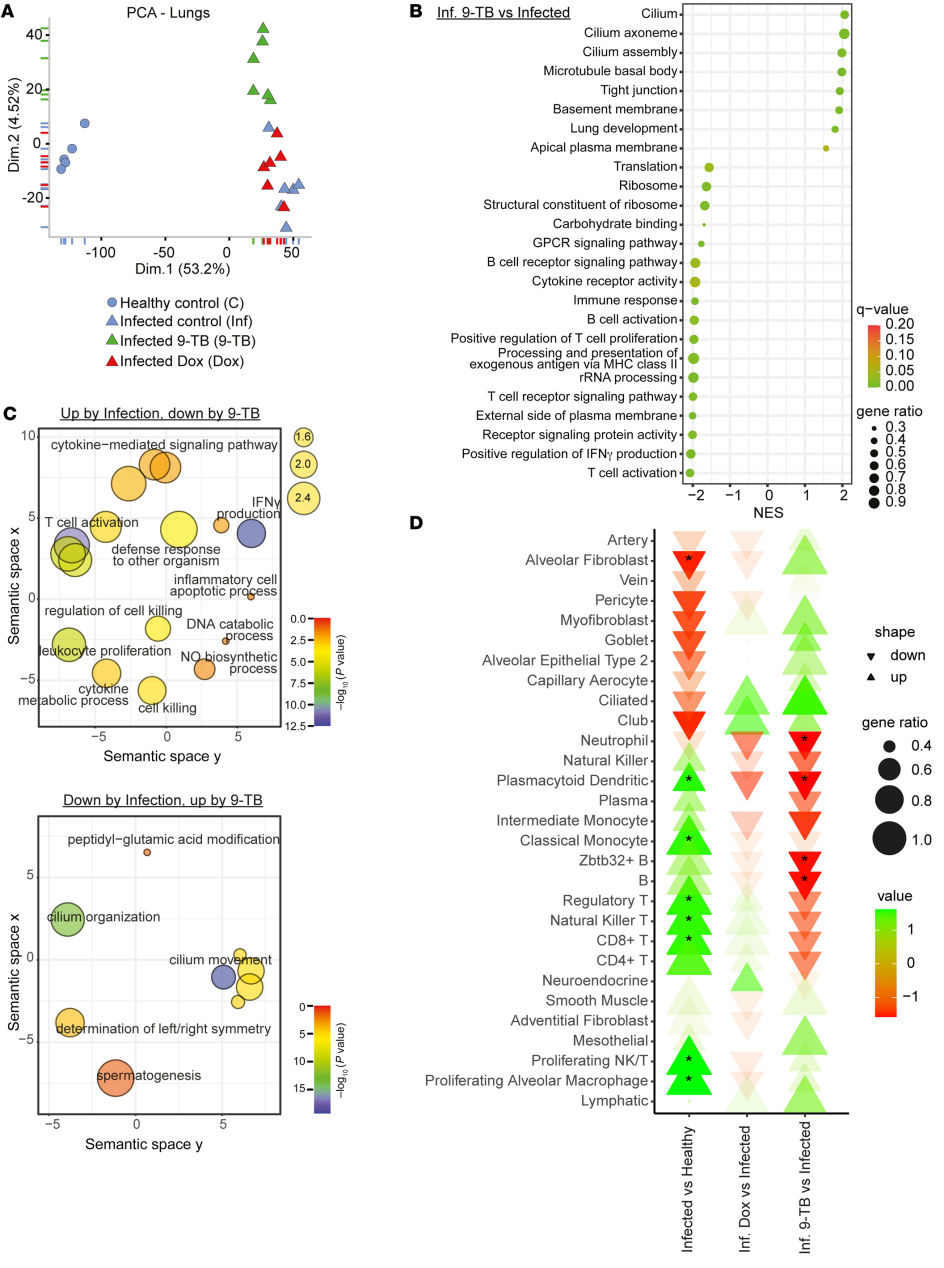
9-TB counteracts the inflammatory and lung-damaging effects of IFV infection. (**A**) Principal component analysis (PCA) of lung RNA-Seq transcriptomes collected on day 7 after infection of BALB/cN mice with 175 PFU IFV H1N1 PR8 (*n =* 5–6). (**B**) Gene set enrichment analysis (GSEA) results for Gene Ontology (GO) gene sets modulated in the comparison between 9-TB–treated versus control IFV-infected mice. A positive normalized enrichment score (NES) corresponds to an overall upregulation, while a negative NES indicates downregulation, of the corresponding gene set. (**C**) Revigo plot summarizing the main themes in the significantly enriched GO Biological Process (GOBP) sets among genes induced by IFV infection and downregulated by 9-TB (left panel), and genes downregulated by IFV infection and induced by 9-TB (right panel). The size of the bubbles (top right legend) is proportional to the number of annotations for the GO term (i.e., frequency) in the GO annotation database, with more general terms displaying larger bubbles. (**D**) GSEA results of the RNA-Seq data showing the directionality (increase or decrease) of the modulated lung cell transcript profiles based on common markers shared by both human and mouse lung cell types derived from extant single-cell transcriptomic data (43). The α value (transparency) represents the –log_10_(adjusted *P* value) of the enrichment. *Adjusted *P* < 0.05.

To gain insight into the mechanisms underlying the Tet-induced disease tolerance, we analyzed the transcriptome of the lung, as well as that of the liver and kidney (Supplemental Figure 6A), 2 organs often affected by the multiorgan failure syndrome seen after infection by respiratory viruses like IFV or SARS-CoV-2 (40). In each tissue, principal component analysis (PCA) separated noninfected from IFV-infected mice along the first dimension, PC1, whereas 9-TB had a more pronounced and less variable effect, with better clustering and further separation, along the second dimension relative to control and Dox transcriptomes (Figure 6A and Supplemental Figure 6B).

GSEA showed that 9-TB significantly downregulated multiple inflammatory and immune-related terms in the lungs (Figure 6B and Supplemental Figure 6D), such as “immune response,” “T cell activation,” or “B cell activation.” We then sought to characterize how 9-TB reversed the effect of IFV infection on transcript levels (Supplemental Figure 6A). We thus assessed which Gene Ontology (GO) Biological Processes (GOPB) terms were enriched in the intersection of gene sets changed in opposite directions by infection and 9-TB, respectively (Supplemental Figure 6C). As summarized by Revigo representation (41) (Supplemental Figure 6A), inflammatory, immune, and apoptotic processes were the main enriched terms among genes induced by IFV and downregulated by 9-TB (Figure 6C). Infection by IFV leads to lung epithelial cell dysfunction and downregulation of genes implicated in cilia or tight junctions, which underpin failures of mucociliary clearance and barrier function that contribute to the pathogenesis of ARDS (19). Accordingly, multiple gene sets related to lung development and to lung cell function and structure were decreased by IFV infection and their expression was restored by 9-TB (Figure 6B, Supplemental Figure 6E, and Figure 6C). Altogether, the results show that 9-TB elicits disease tolerance to IFV mainly by counteracting inflammation and the loss of lung epithelial cells and structures, processes that directly determine the severity of infection by respiratory viruses.

To estimate the impact of the infectious challenge or Tet treatment on the lung cell types, we used single-cell RNA sequencing (scRNA-Seq) transcriptomic profiles of mouse (42) and human (43) lung cell populations from 2 independent studies studying IFV infection; one of these studies furthermore established cell markers that overlap between mouse and human lung cell types (43). Using these cell profiles to perform GSEA on our data confirmed that 9-TB reverted the loss of multiple cell types crucial to lung function, such as club, ciliated, and alveolar epithelial cells (Figure 6D), whereas it decreased several classes of immune cells, such as neutrophils, natural killer cells, and monocytes, all contributing to tissue damage upon IFV infection (17). Dox showed similar, but more discrete, tendencies toward changes in cellular patterns (Figure 6D). These observations were confirmed when using a different set of scRNA-Seq profiles of IFV-infected mouse lungs (Supplemental Figure 7A) (42).

9-TB also downregulated multiple immune-related and inflammatory gene sets in liver and kidney (Supplemental Figure 7B). In particular, as shown through Revigo analysis, these immune and inflammatory terms were enriched among the group of liver genes induced by IFV and downregulated by 9-TB (Supplemental Figure 7, C and D). In the 3 organs studied, Dox led to a weaker downregulation of many of these terms, as shown by GSEA (Supplemental Figure 7B), suggesting that it does not lower systemic IFV-driven inflammation as efficiently as 9-TB, which is also consistent with its more moderate impact on IL-6 plasma levels (Figure 4D). Furthermore, Dox induced gene sets involved in cytopathic processes and fibrogenesis in liver and kidney (Supplemental Figure 7B), suggesting an improved safety profile of 9-TB, relative to Dox, at doses showing similar efficacy. Further investigations will be needed to establish whether the relative upregulation of extracellular matrix/collagen gene sets by 9-TB in lungs (i.e., the site of highest tissue damage due to the infectious challenge) corresponds to proper healing and tissue repair mechanisms (Supplemental Figure 7). Taken together, our transcriptomic data highlight that Tet-induced mitohormesis (and in particular 9-TB) elicits disease tolerance to IFV by preventing IFV-associated lung damage and by dampening inflammatory responses not only in lungs, but in liver and kidney as well.

## Discussion

Here we report that Tets can be used to safely induce an adaptive mitohormetic response, leading not only to the activation of the ATF4/ISR pathway, but also to the induction of type I IFN signaling in vitro and in germ-free mice. This translates into a beneficial impact on lethal IFV infection, where Tets enabled survival and induced disease tolerance of infected mice. RNA-Seq data from lung, liver, and kidney helped to unveil the mechanisms underlying tolerance to IFV infection. Tets rescued the transcript levels of genes involved in lung epithelial cell function and implicated in cilia or tight junctions, which are often found downregulated upon viral respiratory infections as a result of lung damage (19). In contrast, Tets systematically downregulated multiple inflammatory and immune-related gene sets in the lung, liver, and kidney transcriptome data (Figure 6, B and C, and Supplemental Figure 7, B and D). Enrichment of cell profiles based on scRNA-Seq suggested that Tets attenuated the loss of club, ciliated, and alveolar epithelial cells in the lungs, while reducing immune cell infiltration (Figure 6D). We also ascertained a robust reduction of IL-6 (Figure 4, D and H) and of other markers of inflammation and tissue damage (Supplemental Figure 4D). We furthermore demonstrate that non-antimicrobial Tets, such as 9-TB, do not cause disturbances of the microbiome upon treatment in vivo in mice as shown by profiling bacterial species in longitudinally collected feces (Figure 5, A and B). We finally provide highly encouraging results suggesting that even 9-TB administered therapeutically 1 day after inoculation with IFV can increase survival, supporting the clinical relevance of the study. Although the detailed doses and kinetics of the treatments and the immune response to IFV remain to be investigated, we speculate that a possible mechanism for Tet-induced mitohormesis may involve a mild boost of the IFN response early after treatment, leading to overall favorable consequences for inflammation and tissue damage (44), as explained below.

An important limitation for the use of Tets, which inhibit both the bacterial and hence also the mitochondrial translation, comes from their antimicrobial activity that influences the host microbiome. Using a primary screening that identified compounds based on the induction of the UPR^mt^ in *C*. *elegans*, combined with the analysis of their antimicrobial activity, we identified a series of candidates through preliminary structure-activity relationships that have no or very weak antimicrobial activity, yet retain full or even superior capacity to induce the MSR. Indeed, lower doses of 9-TB and ATc led to the induction of the MSR in vitro and to disease tolerance in vivo; these compounds were devoid of some of the adverse effects of Dox (Supplemental Figure 7). Moreover, at the doses required, 9-TB had no detectable effect on the composition and diversity of the gut bacterial communities, as assessed by whole-metagenome sequencing in longitudinally collected mouse feces, while Dox did (Figure 5, A and B, and Supplemental Table 8). This confirms that 9-TB can target mitochondria while not affecting commensal bacteria, both ruling out the hypothesis that the observed effects may be partly mediated by a direct impact on the microbiome and providing additional evidence for its target specificity (Figure 5, A and B, and Supplemental Table 8). This also indicates that one can select Tet derivatives for their enhanced activity on the mitobiome versus the microbiome and thereby eliminate some adverse effects of the Tets.

Mild levels of mitochondrial damage or dysfunction have the potential to activate type I IFN or other immune pathways, leading to inflammation (45). We also highlight this inverse relationship between mitochondrial function and type I IFN signaling in Dox-treated livers, kidneys, and BMDMs (Figure 1, A–L, and Supplemental Figure 1, F and G). It hence appears logical that immune and mitochondrial quality control pathways are coregulated in interlocked feedback circuits to prevent mitochondrial damage upon inflammatory triggers and vice versa. In line with this, a similar inverse correlation between mitochondria-encoded genes, a proxy for both mitochondrial content and functionality, and type I IFN genes was reported in multiple cell types (46, 47), including in IFV-infected mouse lung cells analyzed by scRNA-Seq profiling (42). This also indicates that moderate mitochondrial stress could be adaptive and trigger the induction of low levels of type I IFN that are beneficial (48). Indeed, a finely tuned IFN response allows a balanced immune response with optimal protection and minimal tissue damage, limiting the detrimental effects of a persistent IFN response (49). As an example, in COVID-19, endogenous high levels of type I IFN are protective (50) and early administration of IFN-α decreases mortality, while late administration of IFN-α increases mortality (51); this is consistent with the fact that delayed or chronic IFN responses disrupt lung repair and induce immunopathology (52, 53), while early administration of type I IFN is protective in IFV and coronavirus infections (49, 53). Deciphering exactly how such a moderate type I IFN response is coupled to a beneficial effect on inflammatory status and disease progression is thus particularly challenging given the dual nature of the immunomodulatory functions of type I IFN. Future investigations will have to determine how mitochondrial quality control and innate immune pathways mechanistically combine to translate into Tet-induced disease tolerance to IFV and potentially other viral infections. The ensuing insight may not only open new therapeutic avenues to cope with infections by respiratory viruses, but also to manage other diseases typified by mitochondrial dysfunction and inflammation, such as neurodegenerative (e.g., Alzheimer’s; ref. 54) and cardiovascular diseases (e.g., aortic aneurism; ref. 55).

## Methods

### Mouse experiments in C57BL/6J mice.

Male 9-week-old C57BL/6J mice were treated for 16 days with 500 mpkd Dox hyclate (Sigma-Aldrich) in drinking water. All animals used in the experiments were randomly assigned to experimental or control groups. Mice were housed with ad libitum access to water and food and kept under a 12-hour dark/12-hour light cycle. As doxycycline is bitter, we supplemented the water for both conditions (treatments and controls) with 50 g/L sucrose. Drinking water was changed every 48 hours. Germ-free C57BL/6J mice were obtained from the Clean Mouse Facility, University of Bern (Bern, Switzerland), and compared with specific pathogen–free C57BL/6J mice from Janvier Labs.

### IFV infection in BALB/cN.

Eight-week-old female mice were inoculated on day 0 with IFV A (influenza A/PR/8/34 [H1N1] originating from ATCC VR-1469) via the intranasal route at 175 PFU/mouse/50 μL, 1000 PFU/mouse/50 μL, or 760 PFU mouse/50 μL, depending on the batch of the virus (Supplemental Figure 4, A and B, and Supplemental Figure 5, A and C), under anesthesia by intraperitoneal injection of anesthetic (30 mg/kg Zoletil 50 + 6 mg/kg xylazine). All animals used in the experiments were randomly assigned to experimental or control groups. Mice in all groups were treated with vehicle (saline) or the indicated concentrations of Dox or 9-TB by intraperitoneal injection from day –7 or day –3, respectively, for the preventive treatment, or from day 1 for the therapeutic treatment until death/sacrifice (Supplemental Figure 3, A and B). Body weight was monitored during the entire study. Body temperature, food intake (daily consumption in each cage), and clinical score were monitored from day 0 until death/sacrifice. For each treatment/control group, 10 to 12 mice were followed for survival and 5 to 6 mice were sacrificed on day 7 (respectively day 5) for blood and organ collection. Blood samples were collected in tubes via cardiac puncture and anticoagulated with K_2_EDTA, and then centrifuged at 7000*g*, 4°C for 10 minutes to obtain plasma samples. Any mouse suffering from 35% or greater body weight loss relative to day 0 was euthanized and counted as dead. Mice were blindly scored on a daily basis as follows: 1 = healthy mouse; 2 = mouse showing signs of malaise, including slight piloerection, slightly changed gait, and increased ambulation; 3 = mouse showing signs of strong piloerection, constricted abdomen, changed gait, and periods of inactivity; 4 = mouse with enhanced characteristics of the previous grade, but showing little activity and becoming moribund; 5 = mouse found dead. This part of the study was performed by WuXi AppTec (Shanghai) Co., Ltd.

### HEK293T culture and OCR.

HEK293T cells were purchased from ATCC and are routinely checked in the laboratory for mycoplasma contamination with the MycoProbe detection kit (R&D Systems). HEK293T cells were grown at 37°C in a humidified atmosphere of 5% CO_2_/95% air in DMEM with 4.5 g/L glucose (Gibco) including 10% FBS (Gibco), 1× nonessential amino acids (Invitrogen), and 5 mM penicillin/streptomycin (Invitrogen). Cells were treated for 24 hours with the indicated doses of compounds 24 hours after seeding. OCR was measured with the XF96 instrument (Seahorse Bioscience) according to the manufacturer’s protocol.

### Isolation and culture of primary murine BMDMs.

BMDMs were isolated from the femurs and tibias of 10-week-old C57BL/6J mice. Cells were plated on bacteriological plastic plates in macrophage growth medium consisting of RPMI-1640 (Invitrogen), 1× HEPES (Invitrogen), 5 mM penicillin/streptomycin (Invitrogen), and 10% heat-inactivated FBS (Gibco) supplemented with 15% L cell–conditioned medium as a source of CSF-1. After 1 day, nonadherent cells were collected, seeded at 8 × 10^5^ cells/mL in bacteriological plates, and grown for 5 more days.

### Western blot.

Tissues and cells were lysed using RIPA buffer (50 mM Tris-HCl pH 7.4, 150 mM NaCl, 1% NP-40, 0.5% Na-deoxycholate, 0.1% SDS, 2 mM EDTA, and 50 mM NaF) supplemented with protease and phosphatase inhibitor cocktails (Roche/Thermo Fisher Scientific). Lysates were incubated on ice and cleared by centrifugation at 18,500*g* for 15 minutes at 4°C. Protein concentration was determined by the Lowry method. Proteins were separated by SDS-PAGE and transferred onto polyvinylidene difluoride membranes. Proteins were detected using commercial antibodies against eIF2α, phospho-eIF2α (both from Cell Signaling Technology), HSP90 (Santa Cruz Biotechnology), ASNS (Atlas antibodies), HSPA9 (Antibodies Online), LONP1 (Sigma-Aldrich), OXPHOS proteins (Total OXPHOS Rodent WB Antibody Cocktail, Abcam), and β-tubulin (Santa Cruz Biotechnology). Samples were analyzed by immunoblotting using standard procedures. See complete unedited blots in the supplemental material.

### Microarray analysis and GSEA.

Total RNA was isolated from flash-frozen and powdered liver and kidney aliquots using TRIzol (Life Technologies). RNA was purified using the RNeasy Mini Kit (Qiagen) in accordance with the manufacturer’s instructions. Microarray analysis was performed using Affymetrix mouse MTA1.0 chips in triplicate for each condition. Microarray data were normalized with the RMA-sketch method of the Affymetrix Expression console and analyzed using the limma R package (56). A Bonferroni-adjusted *P* value of less than 0.05 was used to determine the differentially expressed genes. GSEA was performed using the clusterProfiler package (57). Gene sets in gmt format were obtained from the MSigDB database from the Broad Institute website (http://www.gsea-msigdb.org/gsea/msigdb). For each organ, all expressed genes were ordered by decreasing fold change based on the differential expression analysis upon Dox treatment. We performed 10,000 permutations, a minimum gene set size of 10, and a maximum of 1000.

### RNA-Seq analysis and GSEA analysis.

RNA-Seq analysis was performed with extracted RNA from mouse tissues (lungs, liver, and kidneys) recovered on day 7 after intranasal infection with 175 PFU with IFV (influenza A/PR/8/34 [H1N1] originating from ATCC VR-1469) (*n =* 5–6). RNA was extracted from flash-frozen, powdered tissue aliquots and cleaned using TRIzol reagent followed by Direct-zol-96 RNA kit (Zymo Research). RNA quality was assessed using Fragment Analyzer (Agilent). Total RNA (1 μg) was used for the construction of sequencing libraries. For each sample, 60 million paired-end sequencing reads with a length of 100 bp each were sequenced using DNBseq Eukaryotic-T resequencing (BGI Sequencing). FastQC (58) was used to verify the quality of the reads. No low-quality reads were present and no trimming was needed. Alignment was performed against the mouse genome (CRCm38 mm10 primary assembly and Ensembl release 95 annotation) using STAR (version 2.73a) (59). The obtained STAR gene counts for each alignment were analyzed for differentially expressed genes using the R packages edgeR (version 3.24.3) and limma (version 3.38.3) (60) using a generalized linear model. A threshold of 1 log_2_(fold change) and adjusted *P* value less than 0.05 were considered when identifying the differentially expressed genes. A PCA (61) was used to explore the variability between the different samples. The RUVSeq (version 1.16.1) (62) Bioconductor R package was used to correct for the unwanted variation. We used the clusterProfiler R package to conduct GSEA of GO terms (57). We used a minimum gene set size of 10, a maximum gene set size of 500, and performed 10,000 permutations. We used a gene list ordered by log_2_(fold change) from the differential expression analysis. The clusterProfiler (version 3.17.1) package was used for GSEA and various data representations. ReviGO (41) was used to generate clustering of enrichment analysis results. The UpSetR package (63) was used for multiple-group overlap.

### qRT-PCR.

RNA from cells and tissues was extracted using TRIzol and then reverse transcribed into cDNA by the QuantiTect Reverse Transcription Kit (Qiagen), following the manufacturer’s instructions. The qPCR reactions were performed using the LightCycler 480 II system and SYBR Green qPCR Master Mix (Roche). All results are presented relative to the mean of housekeeping genes (ΔΔC_t_ method). All mRNA expression levels were corrected for expression of the housekeeping gene *36B4* or *Actb* for samples of mouse origin, and *ACTB* for samples of human origin. A list of primers used is available in the supplemental material.

### Origin or synthesis of the screened compounds.

The structure, origin, and synthesis method of the screened compounds are indicated in Supplemental Table 3.

### Quantification of mtDNA released into cytosol.

After 1 hour of treatment with the indicated concentration of Dox, day-6-differentiated BMDMs (a 10-cm cell culture for *n =* 1) were harvested by gentle incubation in Cell Dissociation Buffer (Gibco; 2 minutes at 37°C), harvested in a tube, briefly centrifuged (400*g*, 4 minutes), and rinsed once with PBS. Then, the assessment of cytosolic mtDNA was carried out as described and with the same primers as in Kim et al. (64).

### IFN-β measurement in culture medium.

BMDMs on day 6 of differentiation were treated with the indicated concentrations of drugs in a controlled volume of culture medium for 16 to 24 hours. Culture medium was harvested and was assessed for IFN-β concentration using the VeriKine-HS Mouse Interferon Beta Serum ELISA kit (PBL Assay Science) according to the manufacturer’s instructions.

### Compound screening in C. elegans.

The strain used to assess UPR^mt^ activation was SJ4100 (zcIs13[*hsp-6*:*gfp*]) (25) and was provided by the Caenorhabditis Genetics Center (University of Minnesota). Worms were maintained on nematode growth medium (NGM) agar plates seeded with *E*. *coli* OP50 at 20°C. Compound screening plates were obtained by dissolving each compound at 68 μM (except 9-TB at 17 μM, for which the effect was too strong at higher concentrations) in NGM agar supplemented with carbenicillin (25 mg/L) and IPTG (2 mM) and seeded with HT115 RNAi control bacteria or with *cco-1* RNAi clone F26E4.9. L4 larvae were transferred manually onto the compound screening plates and fluorescence was assessed on day 1 of adulthood (similar exposure time for all images). The screening was performed at 20°C.

### Viral titer.

The lung viral titer was determined by plaque assay and the data are shown as log_10_(plaques/g tissue). The plaque assay was performed with the MDCK cells as follows: MDCK cells were seeded at a density of 2.5 × 10^5^ cells/mL. Lung samples were homogenized with a TissueLyser II (Qiagen) after thawing. After centrifugation, the lung homogenates were serially diluted with infection medium, 10-fold for 6 dilutions, and pipetted into a 6-well plate. After incubation, the cell infection medium was replaced with infection medium containing 0.625% low-melting-point agarose. After fixation with 4% paraformaldehyde, cells were stained with 0.5% crystal violet solution. Plaques were counted visually and the viral titer was calculated as follows: viral titer/g lung tissue = log_10_([plaques/well] × dilution factor × 1000).

### Microbiota initial randomization and feces collection.

The mouse microbiome was normalized across cages using a randomization and bedding mixing procedure. On day –17, 24 mice were randomized into 4 balanced groups to mix littermates: vehicle (healthy control), vehicle (infection control), 9-TB treated, and Dox treated. The bedding of each cage was thereafter not changed for 4 consecutive days. On day –13, roughly half of the soiled bedding (with feces) of each cage was collected and mixed in equal amounts in a sterile container. Mice were then put in clean cages filled with half clean bedding and half pooled beddings. This procedure was repeated at the next cage change on day –8. Fecal samples were then collected on day –4, day 0 (inoculation day and 3 days after Dox/9-TB treatment), and day 3 (3 days after infection and 6 days after Dox/9-TB treatment). Each time, mice were individually caged without bedding for 2 to 4 hours and fresh feces were immediately collected and frozen on dry ice. Mice of the vehicle, 9-TB, and Dox groups were inoculated with IFV on day 0 via the intranasal route at a dose of 665 PFU/mouse/50 μL under general anesthesia by injection of anesthetic (30 mg/kg Zoletil 50 + 6 mg/kg xylazine hydrochloride) on the day of inoculation (day 0). Mice in all groups were treated with vehicle (saline) or the indicated concentrations of Dox/9-TB by daily intraperitoneal injection from day –3 until the end of the experiment.

### Whole-metagenome sequencing.

DNA was extracted using the MagMAX Microbiome Ultra Nucleic Acid Isolation Kit (Thermo Fisher Scientific, A42358) using 100 mg of fecal sample for 800 μL of Lysis Buﬀer. Bead beating was performed for 5 minutes at 50 Hz. Lysate was centrifuged at 14,000*g* for 2 minutes and 400 to 500 μL of supernatant was used in subsequent steps using a KingFisher Flex system (Thermo Fisher Scientific) following the manufacturer’s protocol. Extracted DNA was quantified using the Qubit dsDNA Assay Kit (Thermo Fisher Scientific). The sequencing library was prepared with 100 ng of DNA per sample. Briefly, shearing was performed on a Covaris LE200 system, and end repair, A-tailing, ligation of adaptors, and PCR were performed using the KAPA Hyper Prep Kit (Roche, 07962363001) with the following PCR program: 45 minutes at 98°C; 7 cycles of 15 minutes at 98°C, 30 minutes at 60°C, and 30 minutes at 72°C; and then finally 60 minutes at 72°C and holding at 4°C until sample retrieval. Library concentration was measured using the Qubit dsDNA Assay Kit (Thermo Fisher Scientific) and fragment length was assessed on an Agilent TapeStation. The library was sequenced on an Illumina NovaSeq 6000 platform using paired-end 2 × 150 bp chemistry. Sequences were deposited in the European nucleotide archive (ENA) and are publicly available under accession number PRJEB52004.

### Whole-metagenome sequencing data analysis.

Low-quality bases and adapters were trimmed. Short reads (length <35 bp) and low-quality reads were removed. Host sequences were identified by mapping to the host reference genome with bowtie 2 (65), and then removed. Taxonomy was assigned using the the Kraken 2 (66) sequence classifier with an in-house-developed microbial database including 27,165 reference genomes (spanning 9,471 bacteria, 1,854 fungi, 15,752 viruses, and 88 parasites). Genus and species relative abundances in terms of reads per million (RPM) were estimated using Bracken (67). Statistical analysis of the fecal bacterial communities was performed in R and Rstudio. The entire code used in this analysis is publicly available in the GitHub repository (https://github.com/auwerxlab/dox-9tb-mouse-metagenomic-analysis-01) and was archived in Zenodo (10.5281/zenodo.6759368). Briefly, the species composition of the bacterial communities was assessed using perMANOVA based on the Bray-Curtis dissimilarity and 10,000 permutations with *P* values adjusted for multiple comparisons using the Benjamini-Hochberg method. Sample similarities were further assessed using NMDS analysis based on the Bray-Curtis dissimilarity. Bacterial species diversity was assessed in terms of SDI and richness and compared using Kruskal-Wallis 1-way ANOVA followed by Wilcoxon’s post hoc test with *P* values adjusted for multiple comparison using the Holm-Bonferroni method.

### Statistics.

Differences between 2 groups were assessed using 2-tailed *t* tests. Differences between more than 2 groups were assessed with 1-way ANOVA, unless stated otherwise. For survival curves, statistical analysis was performed by log-rank (Mantel-Cox) test. GraphPad Prism 6 was used for statistical analyses. Variability in plots and graphs is presented as standard error of the mean (SEM), unless stated otherwise. All *P* values of 0.05 or less were considered to be significant: *P ≤ 0.05, **P ≤ 0.01, and ***P ≤ 0.001. Mouse experiments were performed once. IFV infection studies and the *C*. *elegans* screening were performed in a blinded manner. Sample sizes for worm, cell, and animal experiments were determined based on previous findings. Sample sizes, replicates, and statistical methods are specified in the figure legends.

### Study approval.

In all studies, animal care was in accordance with institutional guidelines. The germ-free C57BL/6J animal experiments were carried out according to the institutional and national Swiss and EU ethical guidelines and were approved by the local animal experimentation committee of the Canton de Vaud (Service de la consommation et des affaires vétérinaires du Canton de Vaud, Epalinges [Switzerland]; protocol VD2779.a). IFV-infection animal studies were performed according to the protocol following the institutional guidelines of the Institutional Committee Animal Care and Use Committee, Shanghai Site (IACUC-SH; protocol ID01-031-2019v1.1) and approved by the Shanghai Science and Technology Committee (STCSM, Ministry of Science and Technology, PR of China). All animals that showed signs of severe illness, predefined by the animal authorization protocol before the start of the experiment, were euthanized.

### Data and materials availability.

All bioinformatic data associated with the study are present in the paper or the supplemental materials. The data discussed in this publication are deposited in NCBI’s Gene Expression Omnibus and are accessible under GEO Series accession number GSE174124 for RNA-Seq data and under GEO Series accession number GSE202754 for microarray data. Whole metagenome sequences were deposited in the European Nucleotide Archive (ENA) and are publicly available under accession number PRJEB52004.

## Author contributions

The study was conceived and designed by AM, MLN, and JA. AM, TYL, EK, DL, LM, NLH, and DD performed the in vitro, worm, and mouse experiments. MCG and MLN designed, synthesized, and characterized the tetracycline derivatives. AR, GEL, and AM performed the bioinformatics analysis. AM and JA wrote the manuscript with help of MLN, and all authors gave critical comments. JA and MLN supervised the work.

## Supplementary Material

Supplemental data

Supplemental table 1

Supplemental table 2

Supplemental table 3

Supplemental table 4

Supplemental table 5

Supplemental table 6

Supplemental table 7

Supplemental table 8

## Figures and Tables

**Table 1:**
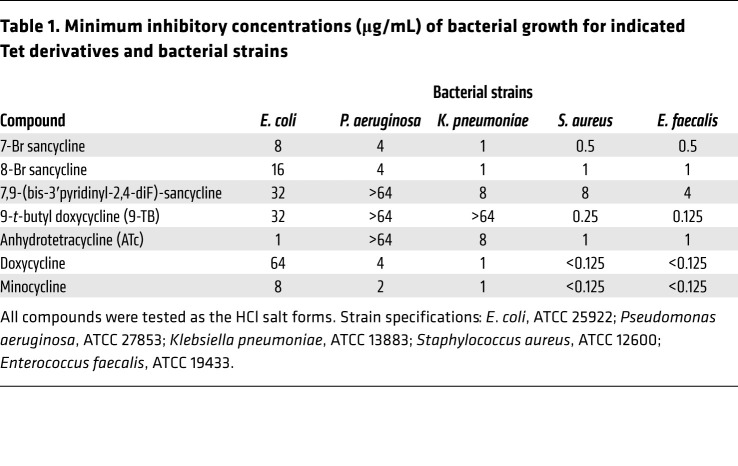
Minimum inhibitory concentrations (μg/mL) of bacterial growth for indicated Tet derivatives and bacterial strains

## Version 1 07/05/2022

In-Press Preview

## Version 2 09/01/2022

Electronic publication
