# Supplementary material for: Tetracycline-induced mitohormesis mediates disease tolerance against influenza
Source: J Clin Invest. 2022 Sep 1;132(17):e151540. doi: 10.1172/JCI151540 (PMC9433105; doi:10.1172/JCI151540)
Supplement: Supplemental data [file jci-132-151540-s008.pdf]

SUPPLEMENTAL MATERIAL

Title: Tetracycline-induced mitohormesis mediates disease tolerance against influenza

Authors: Adrienne Mottis [1], Terytty Yang Li [1], Gaby El Alam [1], Alexis Rapin [1], Elena Katsyuba [1,2], David Liaskos [2], Davide D'Amico[1], Nicola Laraine Harris [3,4], Mark C. Grier [5], Laurent Mouchiroud [2], Mark L. Nelson [5], Johan Auwerx [1,#]

In this document:

Supplementary Methods
Supplementary Figures:
Figure S1: Doxycycline induces the ATF4 and the type I IFN response.
Figure S2: Dose-dependent effects of Tet derivatives on the UPR^{mt}.
Figure S3: Tet derivatives induce MSR genes in mammalian cells.
Figure S4: Tets mediate disease tolerance to IFV in mice.
Figure S5: Tets mediate disease tolerance to IFV in mice. (continued)
Figure S6: 9-TB counteracts the inflammatory and lung damaging effects of IFV infection.
Figure S7: 9-TB counteracts the inflammatory and lung damaging effects of IFV infection. (continued)

Annexes:

Table S1: Differential expression results of liver and kidney microarrays from germ-free mice
Table S2: GSEA results of liver and kidney microarrays from germ-free mice
Table S3: Screened tetracycline derivatives
Table S4: Differential expression results in lungs of IFV-infected mice
Table S5: GSEA results in lungs, liver and kidneys of IFV-infected mice
Table S6: Differential expression results in kidneys and liver of IFV-infected mice
Table S7: Summary of domains relative representation per sample in whole metagenome sequencing reads.
Table S8: Comparison of bacterial community composition by permutational multivariate analysis of variance (perMANOVA)

Supplemental Methods:

RT-qPCR primers

The following primers were used for RT-qPCR (5'→3'):

gene	forward	reverse
**Mouse**		
Asns	TTGACCCGCTGTTTGGAATG	CGCCTTGTGGTTGTAGATTTCAC
Chop/Ddit3	CGGAACCTGAGGAGAGAGTG	CGTTTCCTGGGGATGAGATA
Lonp1	ATGACCGTCCCGGATGTGT	CCTCCACGATCTTGATAAAGCG
Hspa9	AATGAGAGCGCTCCTTGCTG	CTGTTCCCCAGTGCCAGAAC
Hspe1	CTGACAGGTTCAATCTCTCCAC	AGGTGGCATTATGCTTCCAG
Cxcl10	CCACGTGTTGAGATCATTGCC	GAGGCTCTCTGCTGTCCATC
Ifi44	CTGATTACAAAAGAAGACATGACAGAC	AGGCAAAACCAAAGACTCCA
Ddx58	ATGTGCCCCTACTGGTTGTG	CCCCAGAAATGCTCGCAATG
Ifit3	TTC CCA GCA GCA CAG AAA C	AAA TTC CAG GTG AAA TGG CA
Irf7	CAA TTC AGG GGA TCC AGT TG	AGC ATT GCT GAG GCT CAC TT
36B4	AGATTCGGGATATGCTGTTGG	AAAGCCTGGAAGAAGGAGGTC
Actb	GAGACCTTCAACACCCC	GTGGTGGTGAAGCTGTAGCC
Atf4	GAAACCTCATGGGTTCTCCA	GAAAAGGCATCCTCCTTGC
**Human**		
HSPA9	TGGTGAGCGACTTGTTGGAAT	ATTGGAGGCACGGACAATTTT
LONP1	CCCGCGCTTTATCAAGATT	AGAAAGACGCCGACATAAGG
ASNS	ATCACTGTCGGGATGTACCC	TGATAAAAGGCAGCCAATCC

CHOP	ACAGTGTCCCGAAGGAGAAAGG	GCCAAAATCAGAGCTGGAACCT
ACTB	TCGTGCGTGACATTAAGGAG	GTCAGGCAGCTCGTAGCTCT

Complex activity measurements

Starting from powdered tissues, respiratory chain complex activities were measured by Metabiolab (France/Belgium) as described in (*1*). The activities of all the complexes in each sample were normalized by the amount of protein or referred to citrate synthase activity to allow sample comparison.

- Complex I activity measurement

Briefly, reduced nicotinamide adenine dinucleotide phosphate (NADPH)-ubiquinone reductase activity (complex I) was measured by following the disappearance of NADPH using rotenone as a specific inhibitor to ensure the specificity of the assay.

- Complex II activity measurement

Complex II activity, succinate-ubiquinone reductase, was assayed through the reduction of 2,6-dichlorophenolindophenol, a final electron acceptor, after the addition of succinate.`

- Complex III activity measurement

The activity of complex III, ubiquinone-cytochrome c reductase, was determined by assaying the rate of reduction of cytochrome c.

- Complex IV activity measurement

The cytochrome c oxidase (complex IV) activity was based on the same assay as for complex III using potassium cyanide to inhibit its activity.

- Complex V activity measurement

Complex V activity was measured according to a method coupling ADP production to NADH disappearance through the conversion of phosphoenol-pyruvate into pyruvate then into lactate.

- Citrate synthase (CS) activity measurement

The activity of CS was assayed as described previously with the reduction of 5', 5'-Dithiobis 2-nitrobenzoic acid (DTNB) caused by the de-acetylation of acetyl-CoA.

ATP measurement

Total ATP content was measured by the Cell Titer-Glo luminescent cell viability assays (Promega) in protein lysate of kidney powdered tissue. The luminescence was recorded with a Victor X4 plate reader (PerkinElmer) and values were normalized by the total protein concentration determined using a Bradford assay (Biorad).

***C. elegans* experiments in microfluidic system**

UPR^{mt} activation in the nematode *C. elegans* was studied using microfluidic-based testing protocols on an automated platform, as built and optimized by Nagi Bioscience SA (*2*). Briefly, L1 larvae were harvested in complete S-medium and injected into microfluidic chips. Worms were then continuously fed on-chip via bacterial medium, for the duration of the entire experiment. Each tested chemical was added to a 350 µL aliquot of bacterial medium, at 5 concentrations. 4 µL of fresh food/chemical solution were injected into the microfluidic chip per each tested condition every 60 min. Fluorescent pictures of each micro-chamber were acquired after the feeding, every 60 min, during 110 hours. For the fluorescent image acquisition, worms were exposed to blue light for 30 milliseconds. Then, images were analyzed using software algorithms developed by Nagi Bioscience SA, allowing the extraction of the fluorescence intensity per worm. By plotting the average fluorescence intensity over time, the GFP expression kinetic for each condition was plotted. Finally, to quantify the level of GFP expression for each condition, we calculated the Area Under the Curve (A.U.C.) of the mean fluorescence intensity and then extracted the peak fluorescence intensity for each condition. The experiments were performed at 20 °C.

Biochemical parameters measurements

Plasma parameters were measured on 2× diluted samples (1:1 ratio of plasma to kit diluent) using Dimension®Xpand Plus (Siemens Healthcare Diagnostics AG, Dudingen, Switzerland). The biochemical tests were performed according to the manufacturer kit for each parameters: AST (Siemens Healthcare, DF41A), ALT (Siemens Healthcare, DF143), Total protein (Siemens Healthcare, DF73), Urea Nitrogen (Siemens Healthcare, DF21).

Plasma cytokines measurement

Plasma cytokines were measured in plasma using the Luminex Mouse Discovery Assay (R&D Systems) according to the manufacturer's protocol.

Atf4$^{-/-}$ mouse embryonic fibroblasts

Immortalized wild-type and *Atf4*$^{-/-}$ mouse embryonic fibroblasts (MEFs) were kindly provided by D. Ron (Cambridge Institute for Medical Research) (*3*). All cell lines were validated to be free of mycoplasma contamination and maintained in Dulbecco's modified Eagle's medium containing 4.5 g glucose per liter and 10% fetal bovine serum. For culturing the *Atf4*$^{-/-}$ MEFs, 1× non-essential amino acids (Cat. 11140050, Gibco) and 55 μM β-mercaptoethanol (Cat. 31350010, Gibco) were furthermore supplemented to the medium, as described previously (*3*) ; wild-type MEFs were cultured at the same condition for at least one week when comparing with the *Atf4*$^{-/-}$ MEFs. Tetracyclines were dissolved in PBS and added with final concentrations as indicated in the figure legends.

Supplementary figures :

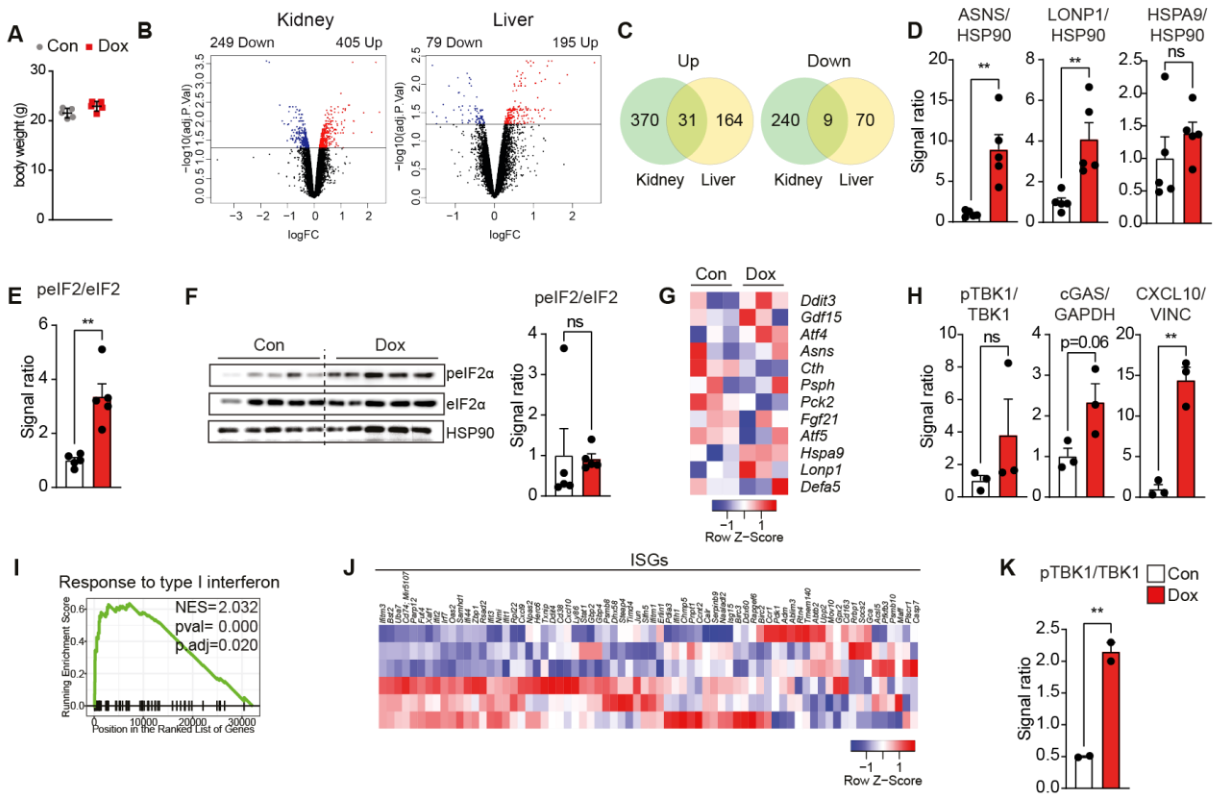

Figure S1: Doxycycline induces the ATF4 and the type I IFN response.

A. Body weight at the time of sacrifice of germ-free C57BL/6J male mice treated with Dox at 500mpkd. **B**. Volcano plots displaying –log10(adj. p-value) and the log fold change in expression of genes in kidney and liver. Above the graph, the number of differentially expressed genes is indicated (genes with adjusted p value (adj.pval)<0.05). **C**. Venn diagrams displaying the genes commonly up and down-regulated in liver and kidney of germ-free mice treated with Dox at 500mpkd (differentially expressed genes with adj.pval<0.05). **D**. Quantification of band intensities of immunoblots in Fig. 1D. **E**. Quantification of band intensities of immunoblots in Fig. 1E. **F.** Immunoblots of phosphorylated eIF2α (peIF2α) and total eIF2α and quantification in liver of Dox-treated germ-free mice. **G**. Heatmap representing the transcript levels of ATF4/5 target genes from liver transcriptomics data of control vs Dox-treated germ-free mice. **H.** Quantification of band intensities of immunoblots in Fig. 1D. **I-J**. Enrichment score plot for the GO term “Response to type I interferon” (I) and heatmap representing the transcript levels of some interferon-stimulated genes (ISGs) (J) from kidneys of germ-free mice treated with Dox. **K**.

Quantification of band intensities of immunoblots in Fig. 1J. Statistical analysis was performed by Student t-test (*p ≤ 0.05, **p ≤ 0.01, ***p ≤ 0.001).

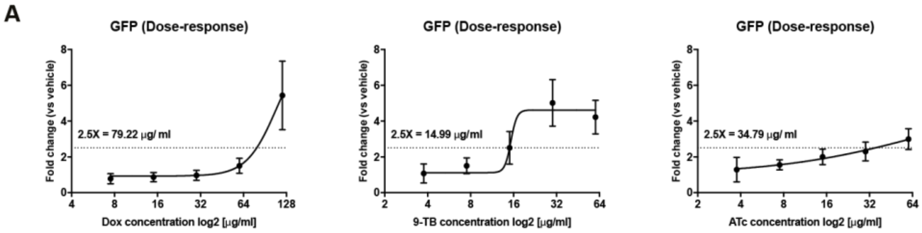

Figure S2: Dose-dependent effects of Tet derivatives on the UPR^{mt} reporter and on development in *C. elegans*.

A. Dose-response curves for the UPR^{mt} activation (*hsp-6::gfp* reporter strain) upon exposure to different concentrations of Dox, 9-TB or ATc using an automated microfluidic device (n=14-16). The efficacy of each drug to induce the UPR^{mt} was evaluated by comparing the concentration where a 2.5-fold change (vs vehicle) in the measured fluorescence intensity was achieved. 9-TB was the most efficacious for surpassing the 2.5-fold change threshold, while Dox was the weakest for inducing the UPR^{mt} at low concentrations. The curve fitting was performed by applying a four-parameter logistic curve. Error bars represent standard deviation (SD).

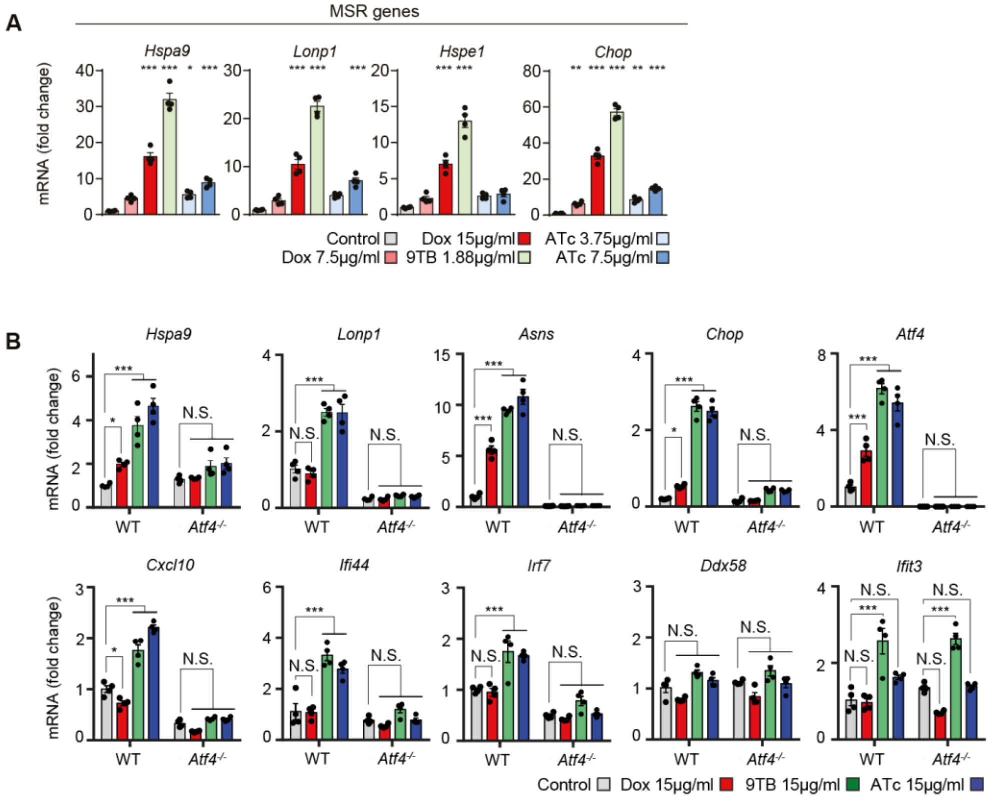

Figure S3: Tet derivatives induce MSR genes in mammalian cells.

A. Transcripts levels of the indicated MSR genes measured by RT-qPCR after 24-hours treatment at the indicated concentrations of the Tet derivatives in mouse bone marrow derived macrophages (day 6 differentiation; n=4). **B**. Transcript levels of the indicated MSR and ISG genes measured by RT-qPCR after 24-hours treatment at the indicated concentrations of the Tet derivatives in WT and Atf4$^{-/-}$ MEFs (n=4). Statistical analysis was performed by ANOVA followed by Tukey post-hoc test (* $p \leq 0.05$, ** $p \leq 0.01$, *** $p \leq 0.001$, N.S: not significant). Error bars represent standard error mean (SEM).

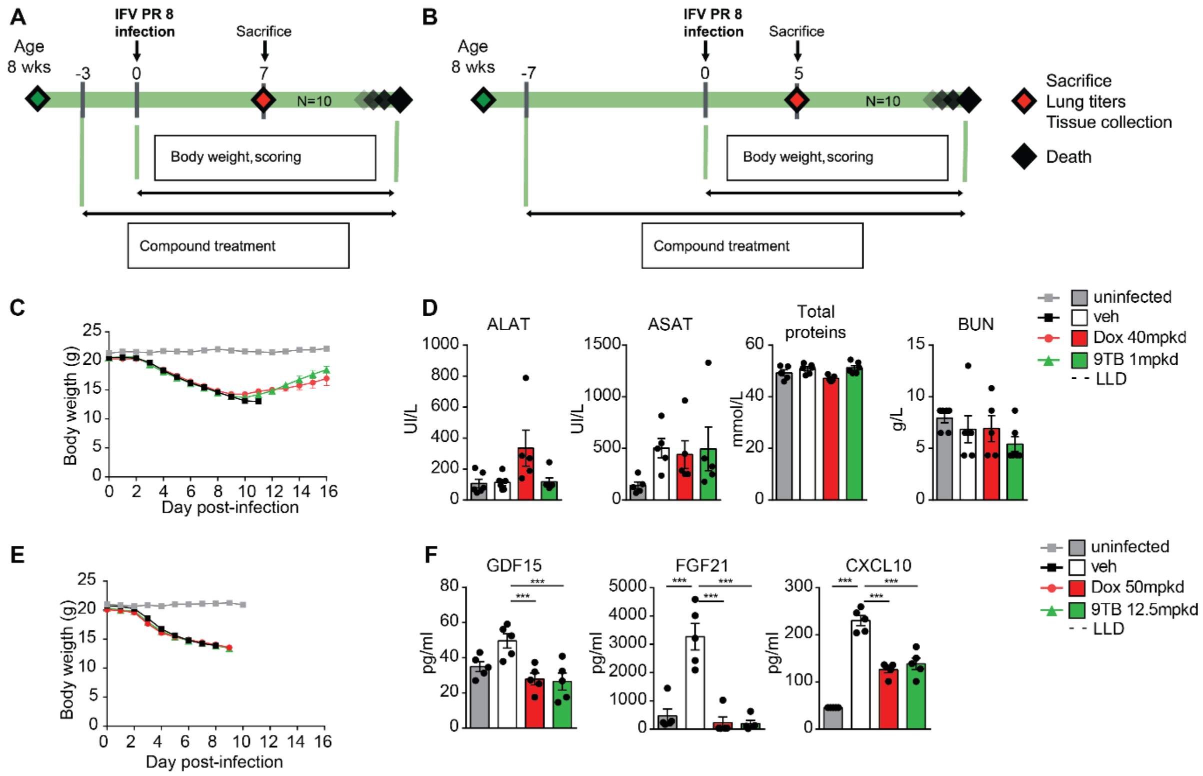

Figure S4: Tets mediate disease tolerance to IFV in mice.

A-B. Descriptive schemes of the 2 respective mouse IFV infection experiments. **C-D**. 8 weeks-old BALB/cN mice (n=15-16) were injected with Dox (40 mpkd) or 9-TB (1 mpkd) and intrasanally infected with 175 PFU of IFV-A H1N1 PR8. **C**. Body weight was followed for 16 days post-inoculation (n=10). **D.** At day 7 post-infection, plasma levels of alanine aminotransferase (ALAT), asparagine aminotransferase (ASAT), total proteins and blood urea nitrogen (BUN) (n=5). **E-F**. 8 weeks-old BALB/cN (n=15-16) mice were injected with Dox (50 mpkd) or 9-TB (12.5 mpkd) and intrasanally infected with 1000 PFU of IFV-A H1N1 PR8, as described in Fig. S3B. **E.** Body weight was followed for 10 days post-inoculation (n=10). **F.** At day 5 post-infection, plasma levels of GDF15, FGF21 and CXCL10 were measured (n=5). Statistical analysis was performed by ANOVA followed by Tukey post-hoc test (**p* ≤ 0.05, ***p* ≤ 0.01, ****p* ≤ 0.001). Error bars represent standard error mean (SEM).

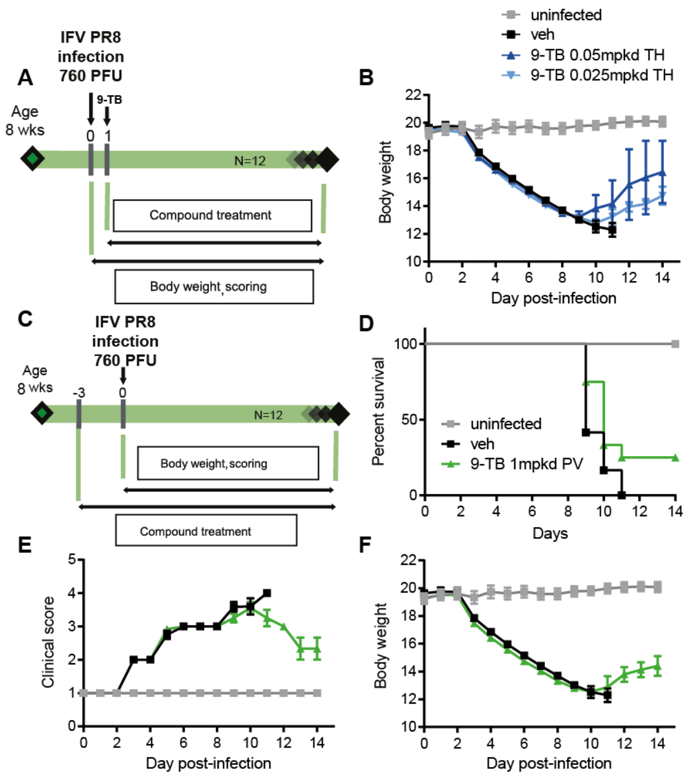

Figure S5: Tets mediate disease tolerance to IFV in mice.

A. Descriptive scheme of the mouse IFV infection experiment depicted in Fig. 5C-D, S5B. **B**. 8-weeks old BALB/cN mice (n=12) were infected intranasally with 760 PFU of IFV-A H1N1 PR8 and injected with 9-TB (0.05, 0.025 mpkd), as described in Fig. S5A. Body weight was followed for 14 days post-inoculation (n=12). **C**. Descriptive scheme of the mouse IFV infection experiment depicted in Fig.S5D-F. **D-F.** 8 weeks-old BALB/cN mice (n=15-16) were injected with 9-TB (1 mpkd) and intrasanally infected with 760 PFU of IFV-A H1N1 PR8. Survival (D), clinical score (E) and body weight (F) were followed for 14 days post-infection (n=12). For survival curves, statistical analysis was performed by Log-rank (Mantel-Cox) test (**p* ≤ 0.05,***p* ≤ 0.01, ****p* ≤ 0.001).

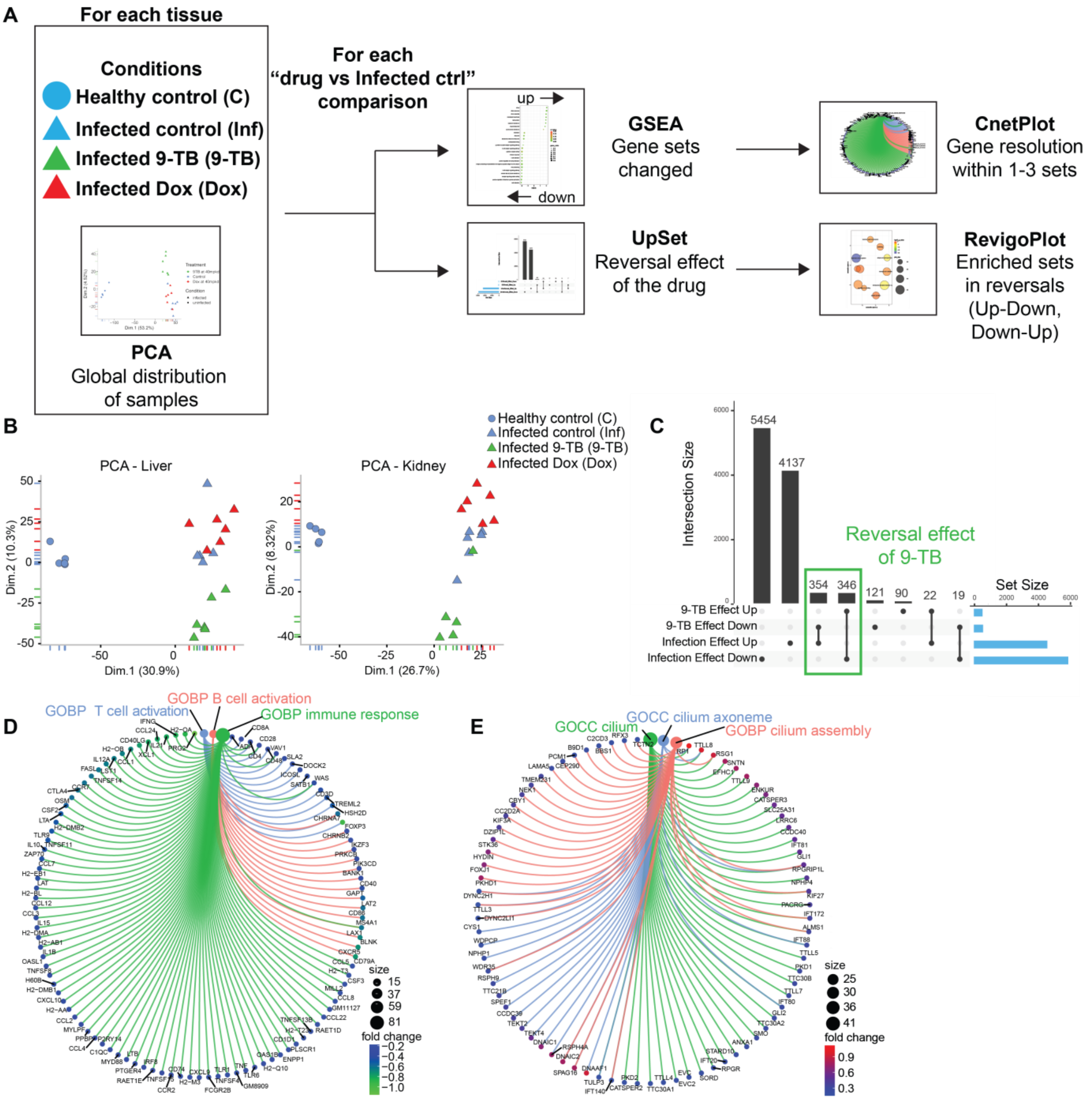

Figure S6: 9-TB counteracts the inflammatory and lung damaging effects of IFV infection.

A. Scheme summarizing the pipeline used for the analysis of transcriptomics data in the tissues. **B**. Principal component analysis (PCA) of liver and kidney transcriptomes collected at day 7 post-infection of BALB/cN mice with 175 PFU IFV-A H1N1 PR8 (n=5-6). **C**. UpSet R plot summarizing the effects of IFV and 9-TB on significantly changed genes in the lung RNA-Seq transcriptomics in 4 different categories (up/down-regulated by IFV/9-TB; with filtering features adj. p-value ≤ 0.05 and absolute fold change ≥ 0.5). A link between 2 dots symbolizes the intersection between the 2 corresponding categories and the corresponding bar right above symbolizes the

number of genes in this intersection. **D-E**. Cnetplots for immune-related (D) and cilium-related (E) gene sets, respectively, representing changes between 9-TB *versus* IFV-infected conditions at transcript level.

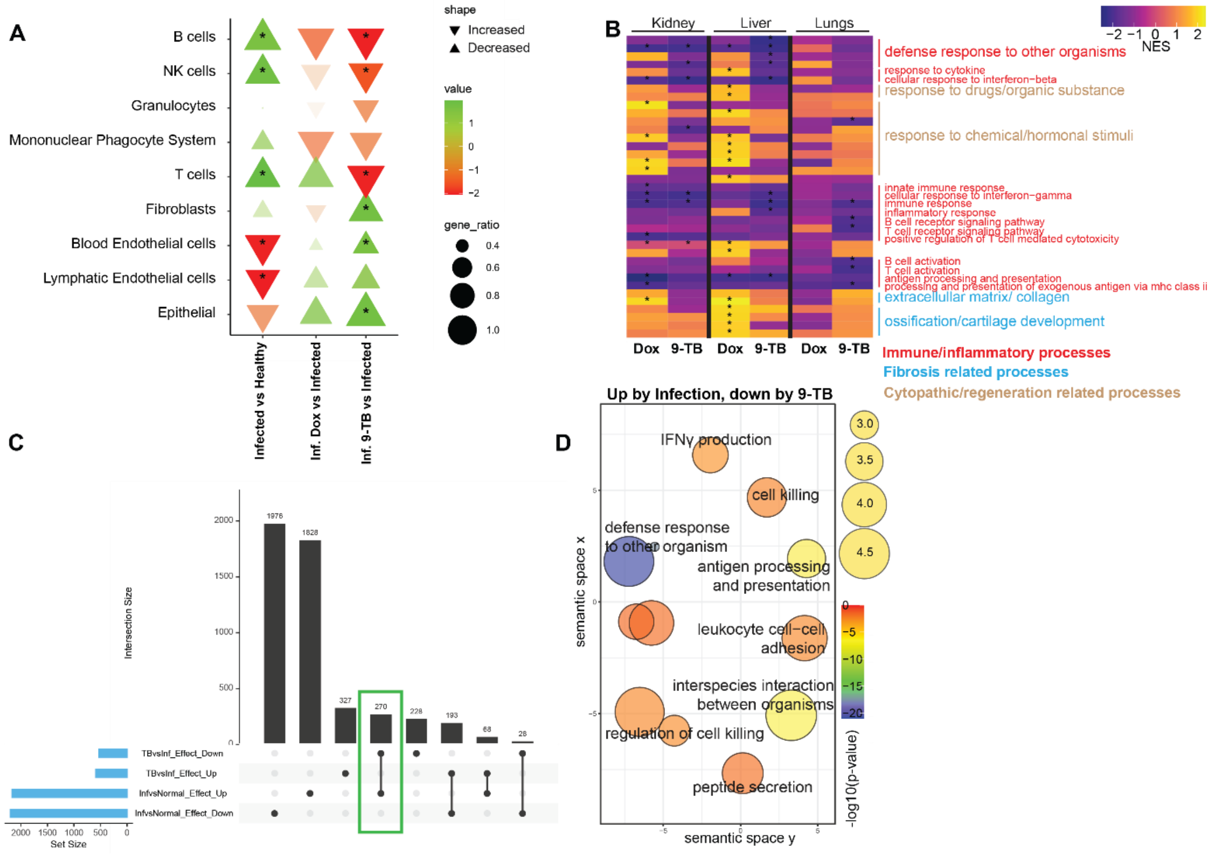

Figure S7: 9-TB counteracts the inflammatory and lung damaging effects of IFV infection. (continued)

A. GSEA results of the RNA-Seq data (Fig. 5) showing the directionality (increase or decrease) of mouse lung cell types described in (*4*). * represents adjusted p-value < 0.05. **B.** Heatmap displaying the GSEA results of chosen gene sets for the effect of 9-TB and Dox compared to the IFV condition. Colour represents the normalized enrichment score (NES) **C.** UpSet R plot of significantly changed genes in the liver (up/down-regulated by IFV/9-TB; with filtering features adj. p-value ≤ 0.05 and absolute fold change ≥ 0.5). A link between 2 dots symbolizes the intersection between the 2 corresponding categories and the corresponding bar right above symbolizes the amount of genes in this intersection. **D.** Revigo plot summarizing the main themes in the significantly enriched GO Biological Process (GOBP) sets amongst genes induced by IFV infection and down-regulated by 9-TB (left panel), as defined in the Upset R plot (Fig. S5C). The size of the bubbles (top right legend) is proportional to the number of annotations for the GO term (i.e. frequency) in the GO annotation database, more general terms displaying larger bubbles.

Bibliography

1. M. Conti, T. Delvienne, S. Loric, in *Mitochondrial Dysfunction Caused by Drugs and Environmental Toxicants*, Y. Will, J. A. Dykens, Eds. (John Wiley & Sons, Inc., Hoboken, NJ, USA, 2018; http://doi.wiley.com/10.1002/9781119329725.ch15), pp. 249–263.

2. H. B. Atakan, M. Cornaglia, L. Mouchiroud, J. Auwerx, M. A. M. Gijs, Automated high-content phenotyping from the first larval stage till the onset of adulthood of the nematode Caenorhabditis elegans. *Lab Chip*. **19**, 120–135 (2018).

3. H. P. Harding, Y. Zhang, H. Zeng, I. Novoa, P. D. Lu, M. Calfon, N. Sadri, C. Yun, B. Popko, R. Paules, D. F. Stojdl, J. C. Bell, T. Hettmann, J. M. Leiden, D. Ron, An integrated stress response regulates amino acid metabolism and resistance to oxidative stress. *Mol Cell*. **11**, 619–633 (2003).

4. Y. Steuerman, M. Cohen, N. Peshes-Yaloz, L. Valadarsky, O. Cohn, E. David, A. Frishberg, L. Mayo, E. Bacharach, I. Amit, I. Gat-Viks, Dissection of Influenza Infection In Vivo by Single-Cell RNA Sequencing. *Cell Systems*. **6**, 679-691.e4 (2018).

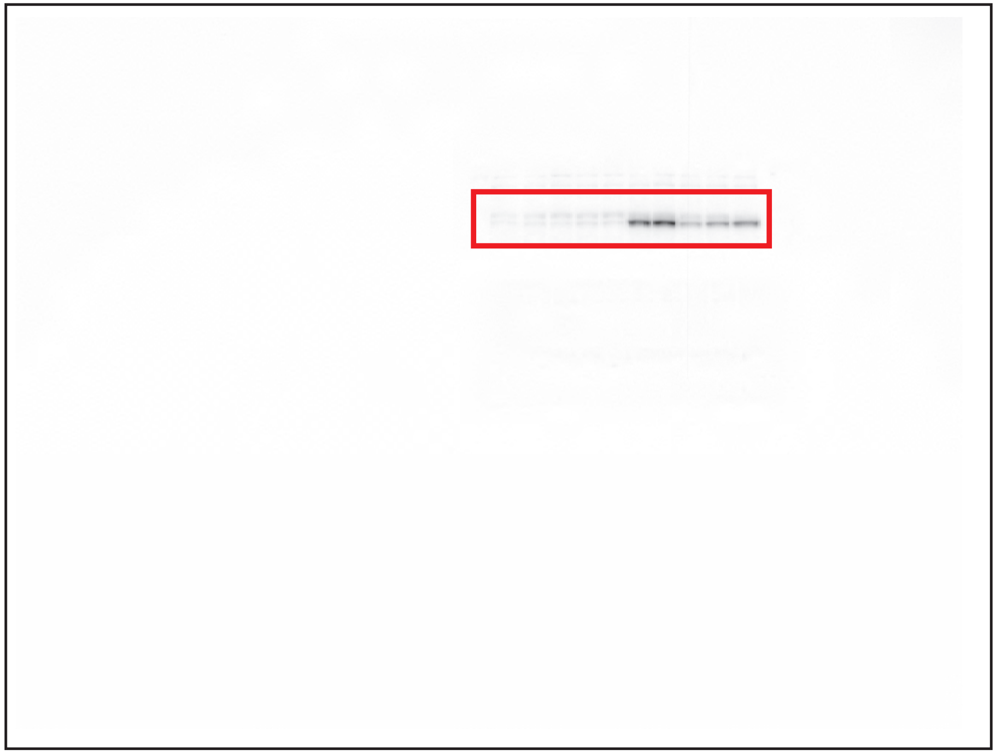

Full unedited gel of Fig1D, ASNS

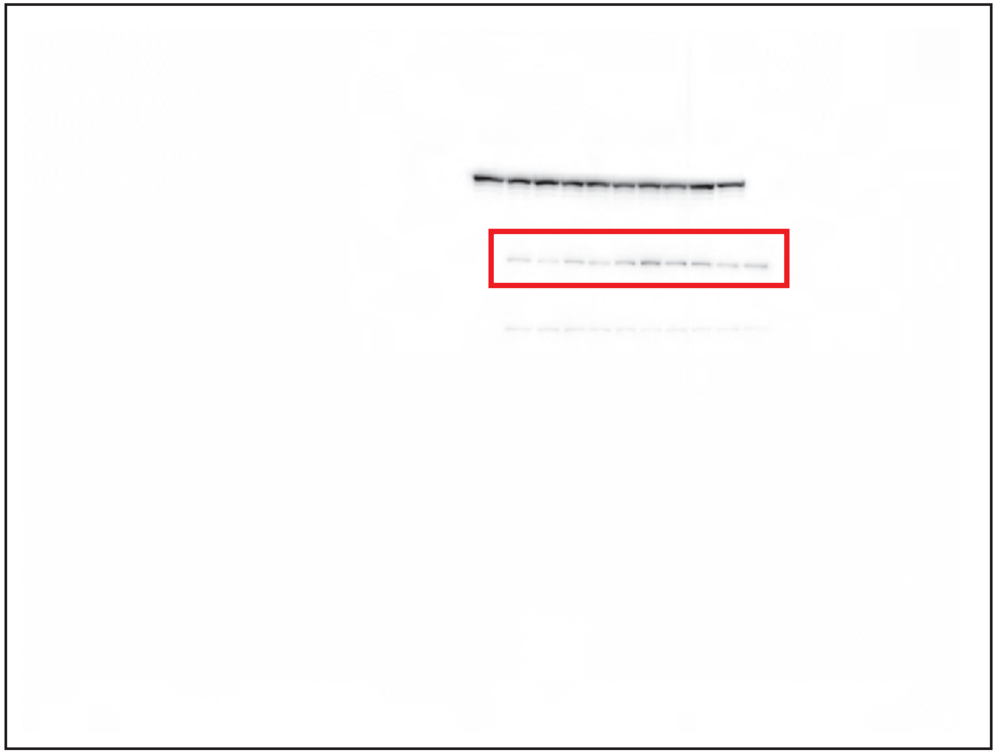

Full unedited gel of Fig1D, LONP1

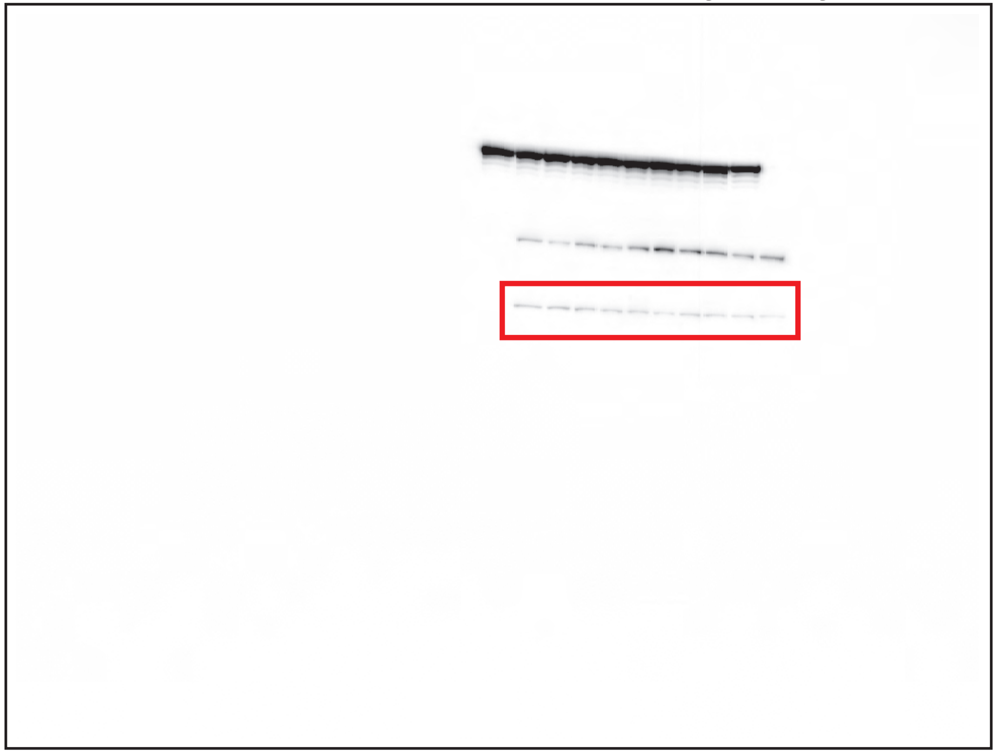

Full unedited gel of Fig1D, HSP90

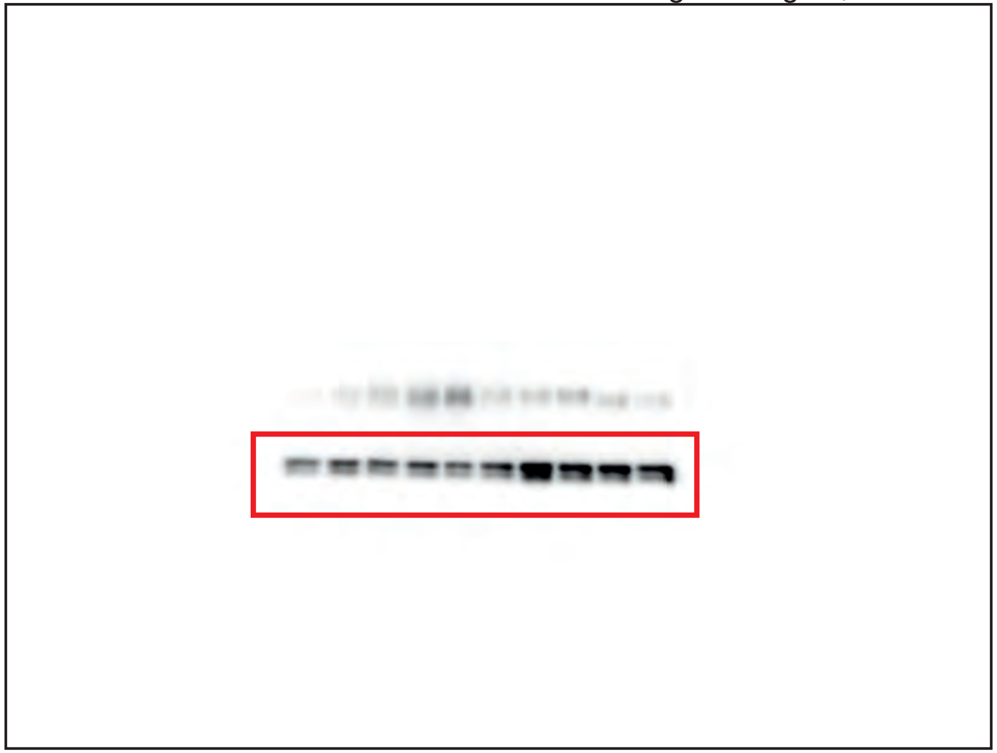

Full unedited gel of Fig1D, HSPA9

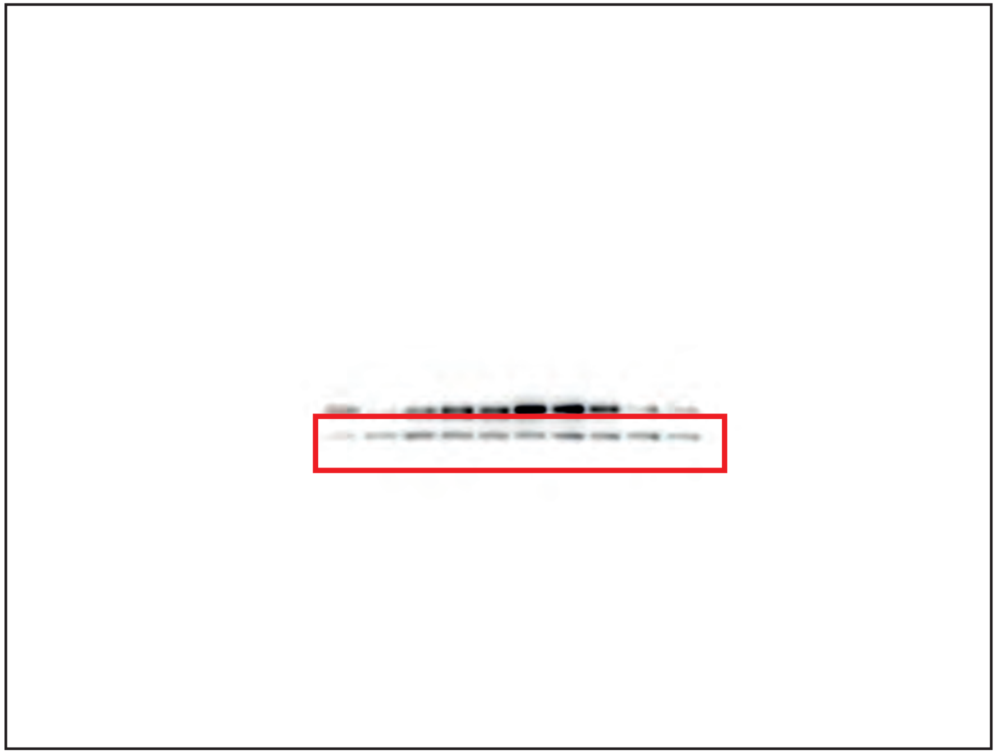

Full unedited gel of Fig1D, HSP90

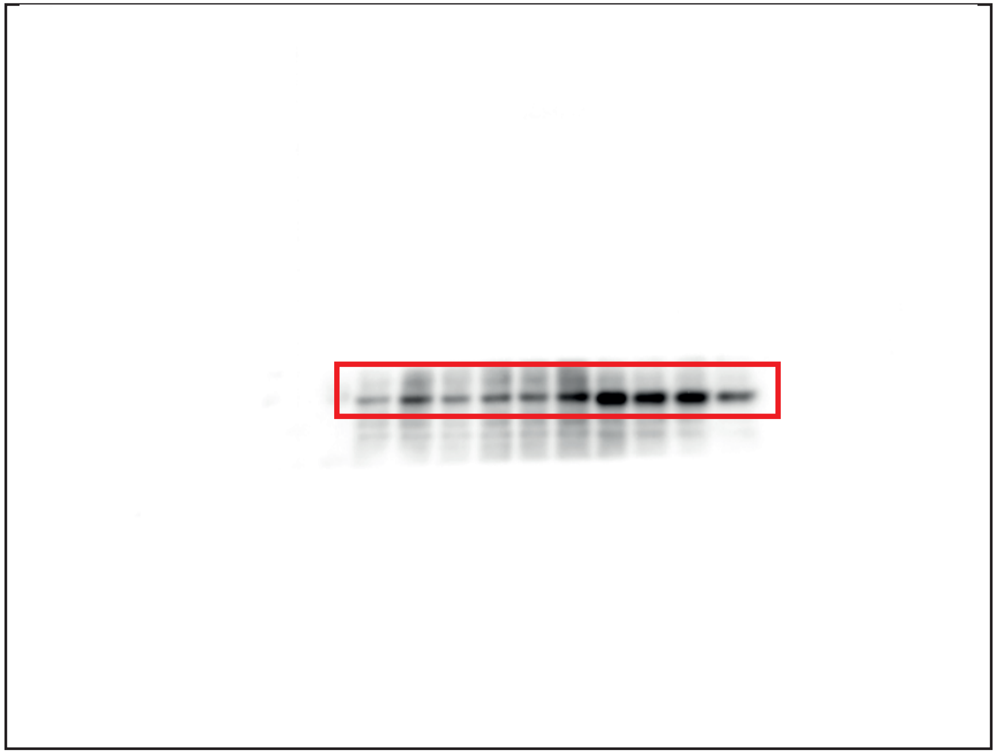

Full unedited gel of Fig1E, pEIF2alpha

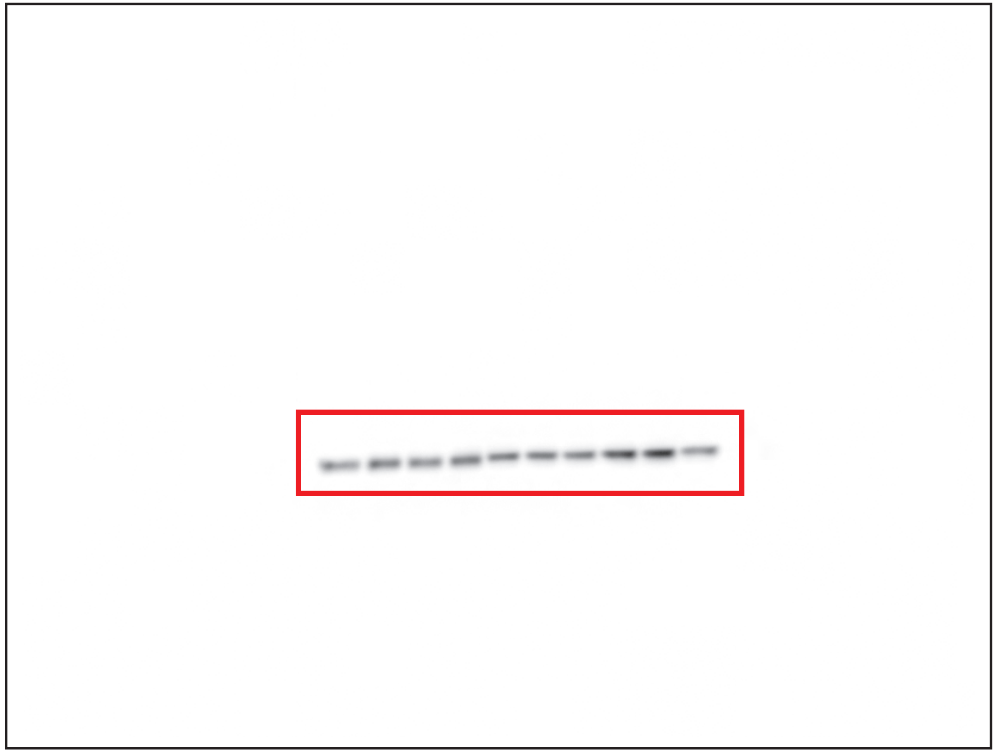

Full unedited gel of Fig1E, HSP90

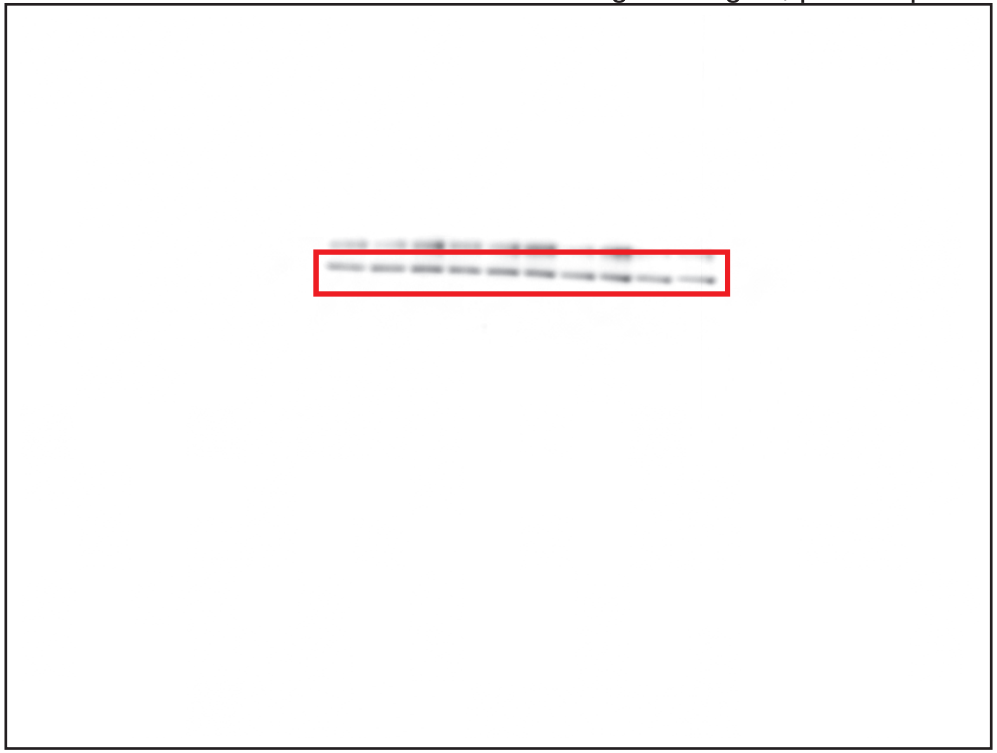

Full unedited gel of Fig1E, HSPA9

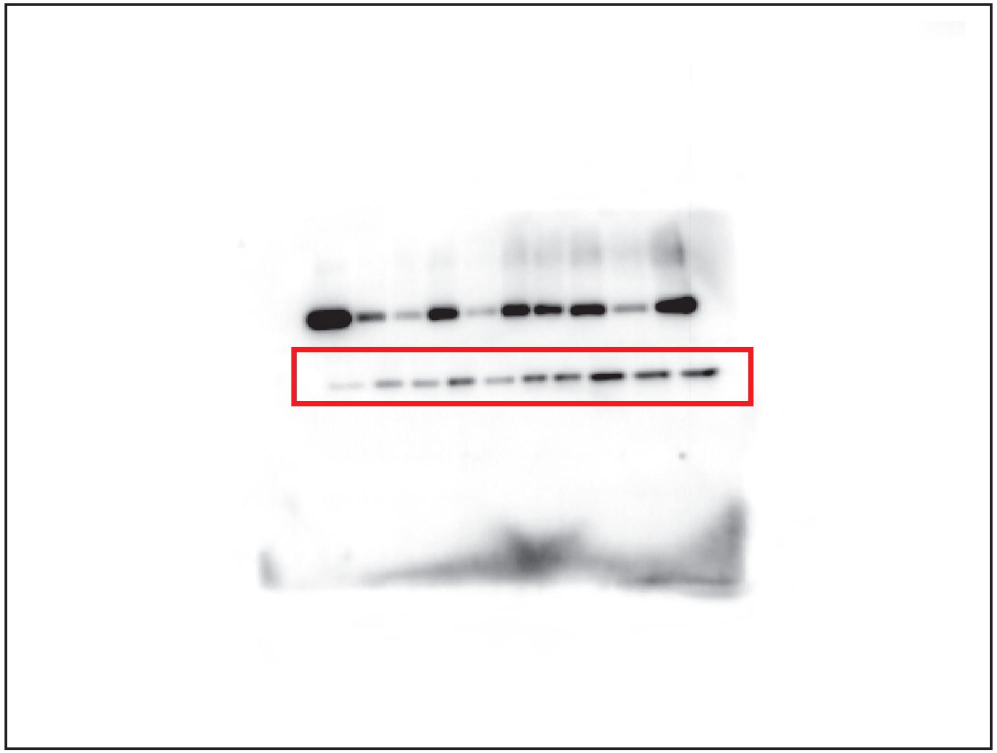

Full unedited gel of FigS1F, pEIF2alpha

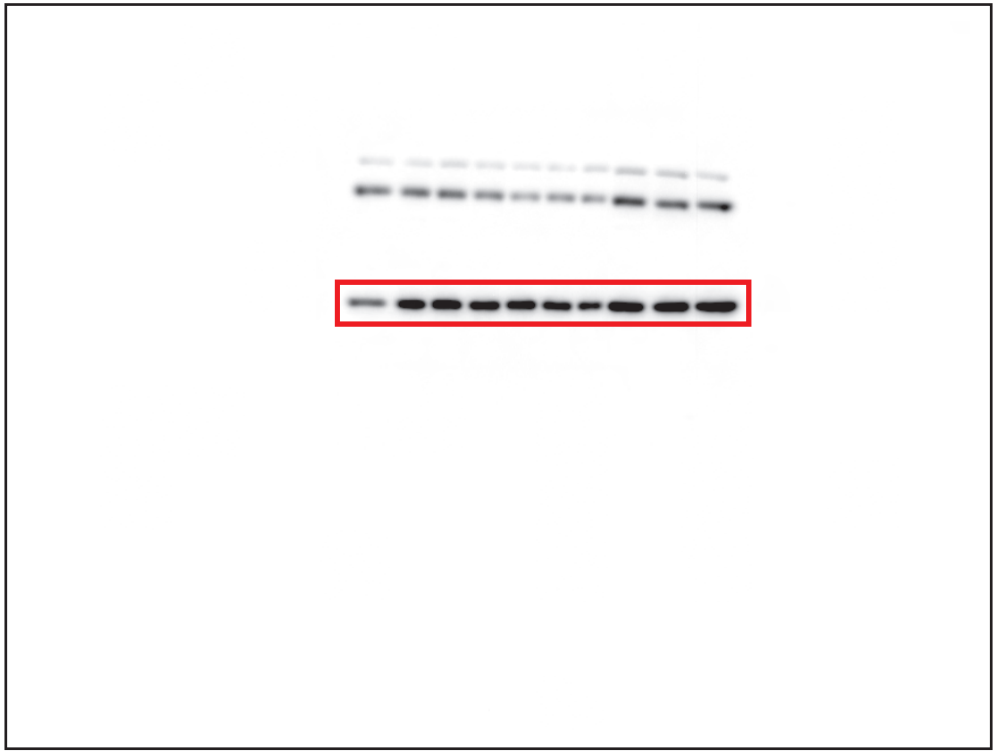

Full unedited gel of FigS1F, EIF2alpha

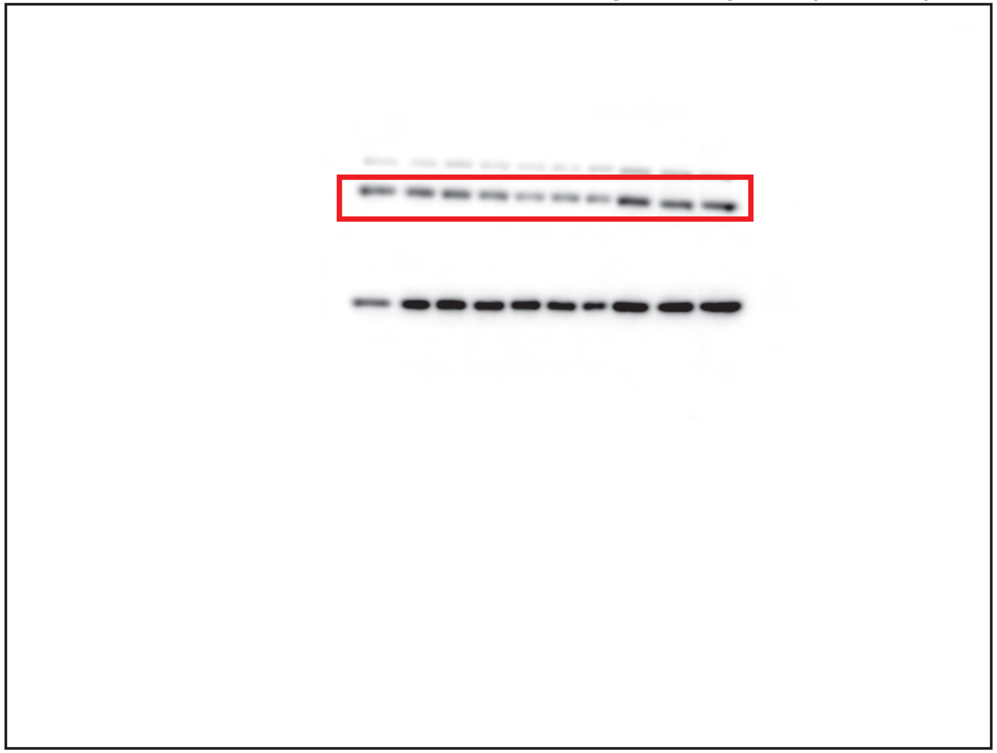

Full unedited gel of FigS1F, HSP90

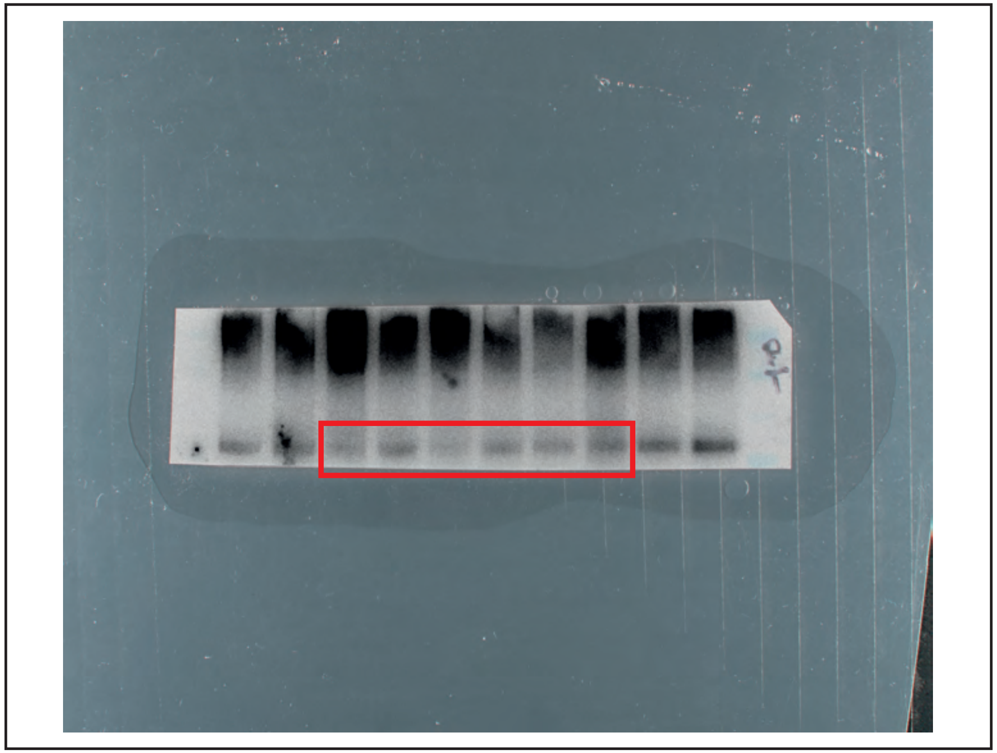
Full unedited gel of Fig1H, pTBK1

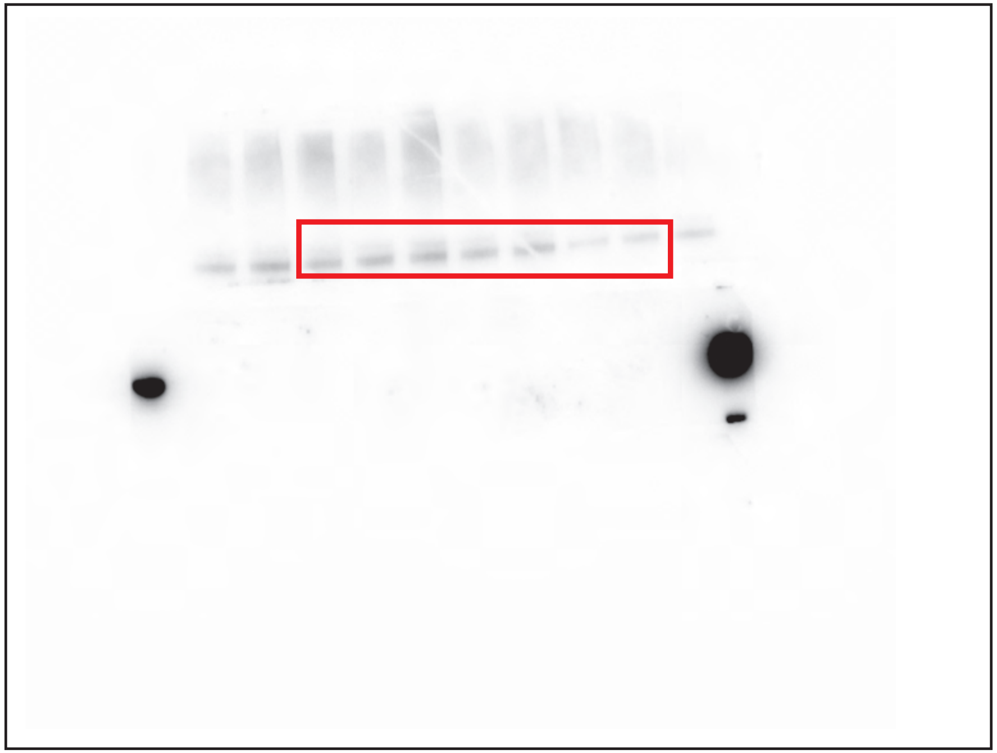
Full unedited gel of Fig1H, TBK1

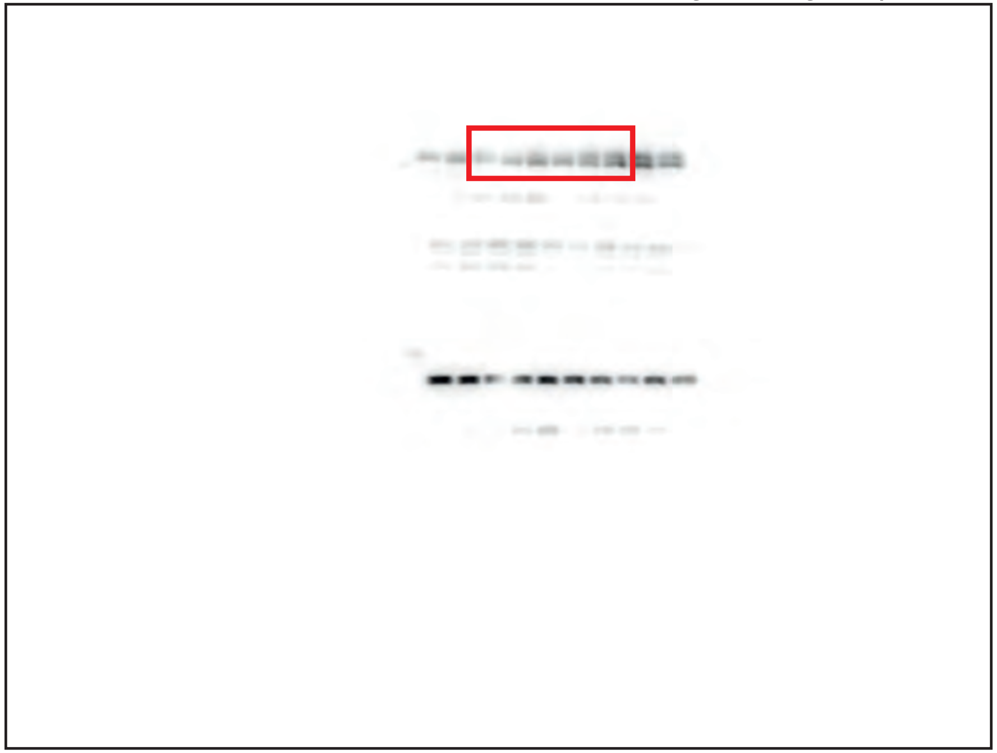
Full unedited gel of Fig1H, cGAS

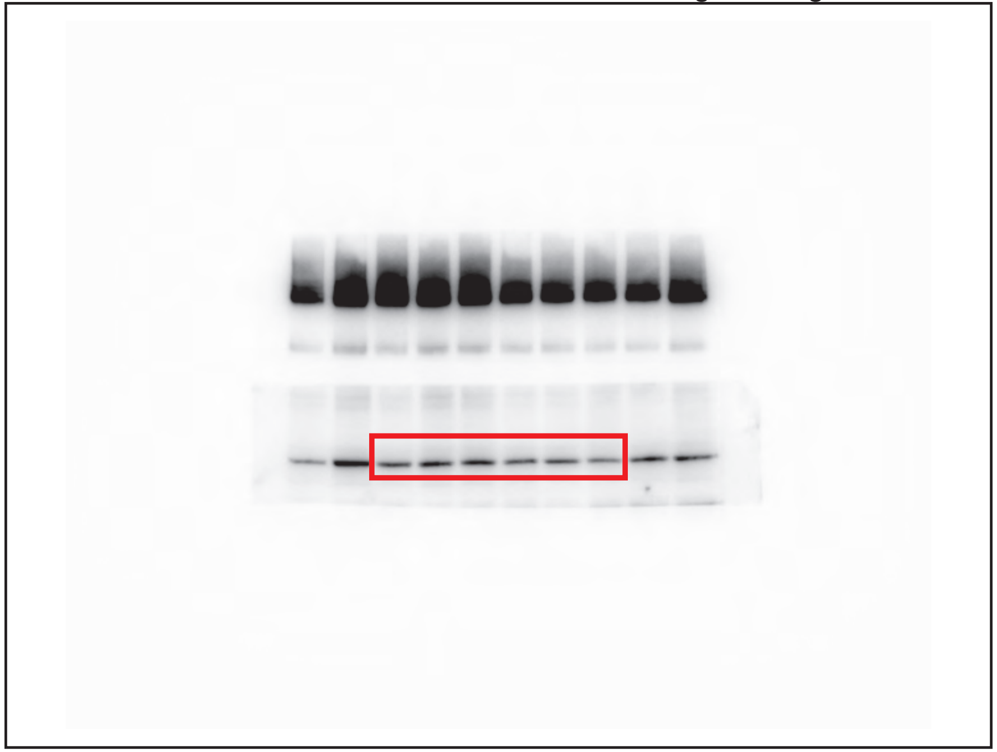
Full unedited gel of Fig1H, GAPDH

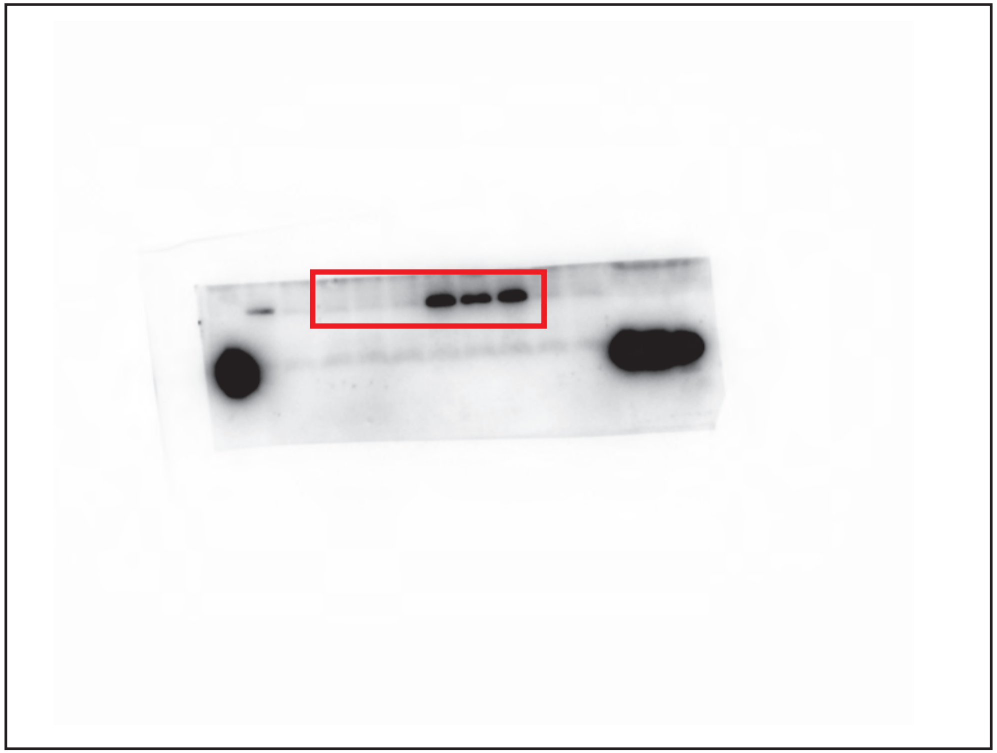

Full unedited gel of Fig1H, CXCL10

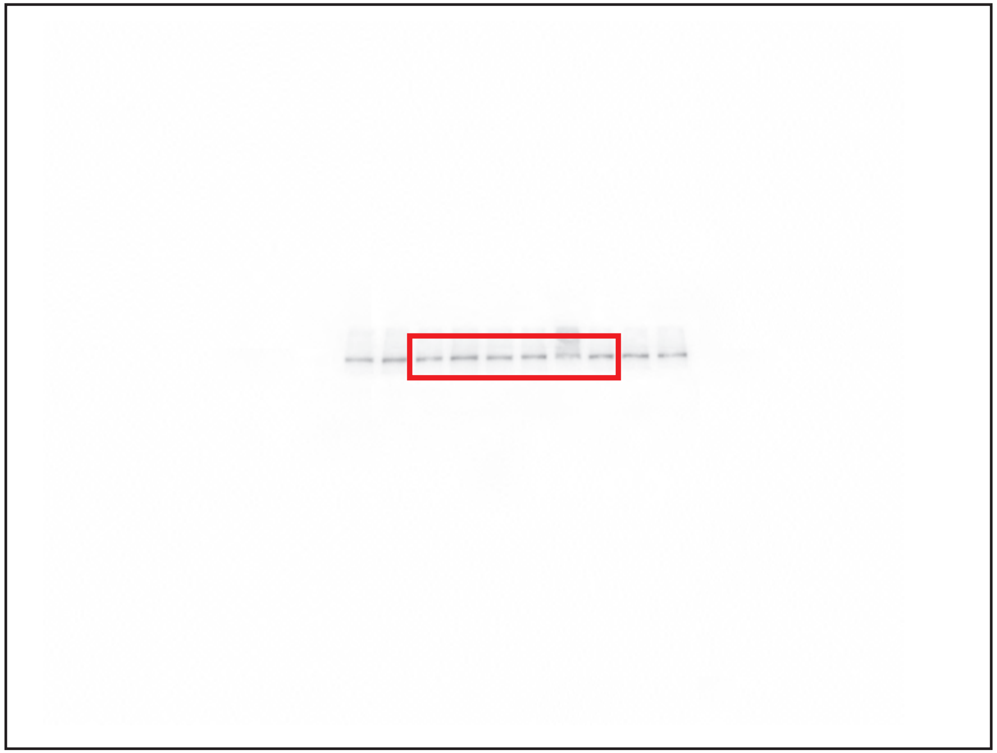

Full unedited gel of Fig1H, VINCULIN

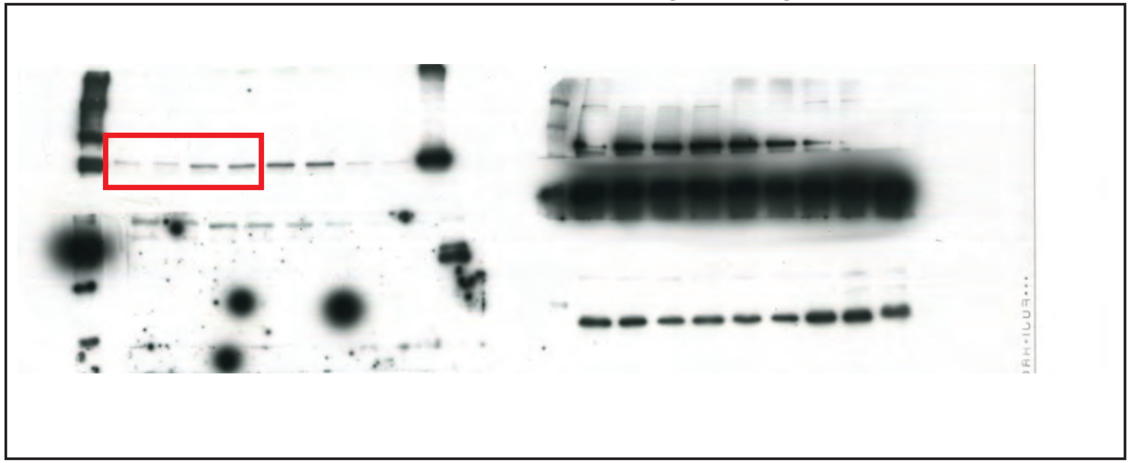

Full unedited gel of Fig1J, pTBK1

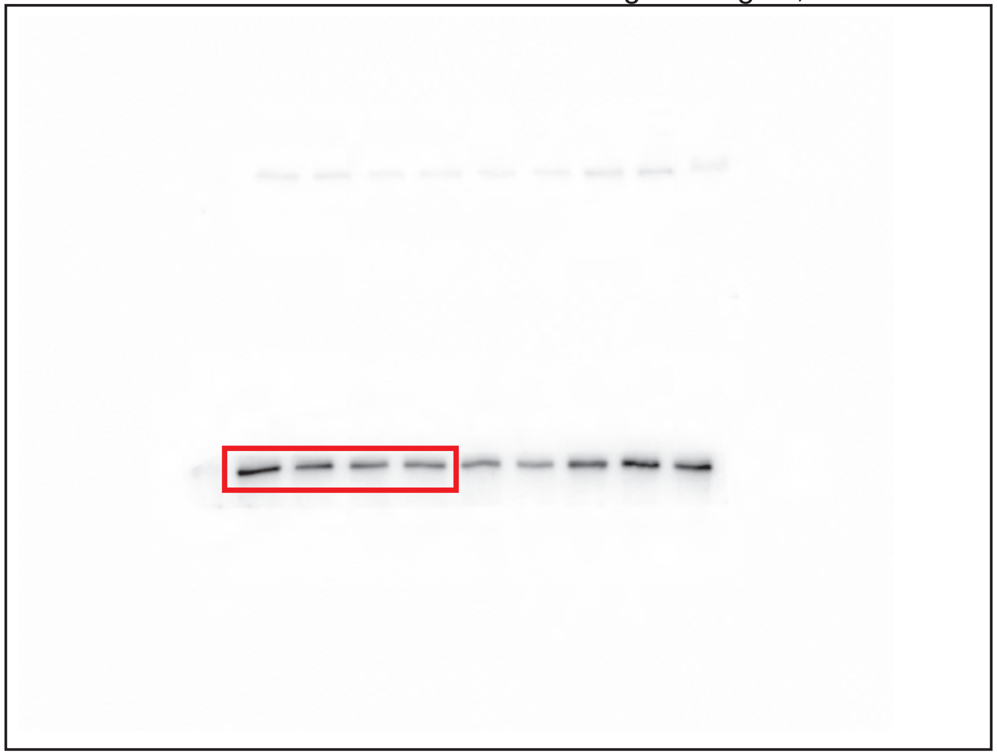

Full unedited gel of Fig1J, TBK1

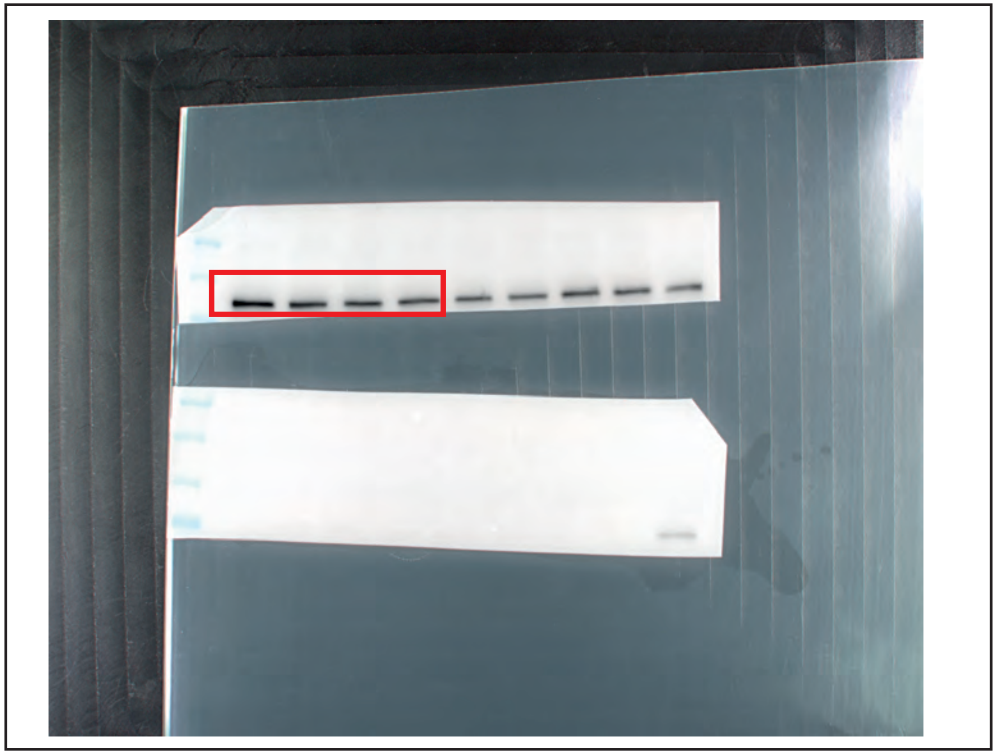

Full unedited gel of Fig1J, VINCULIN

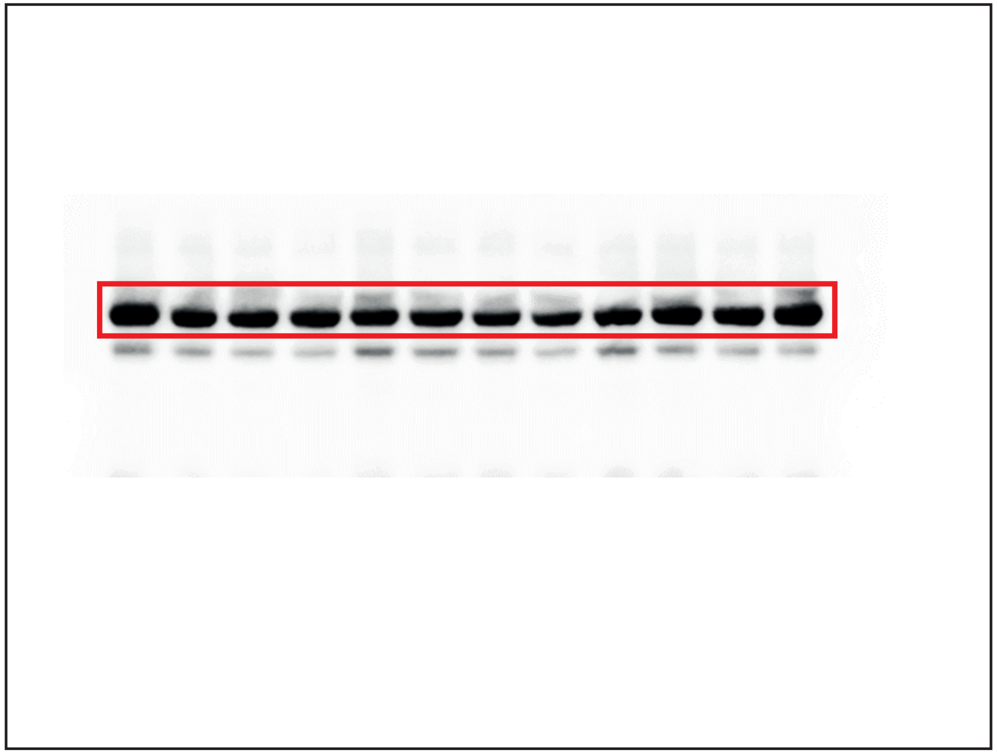

Full unedited gel of Fig3A, ATP5A

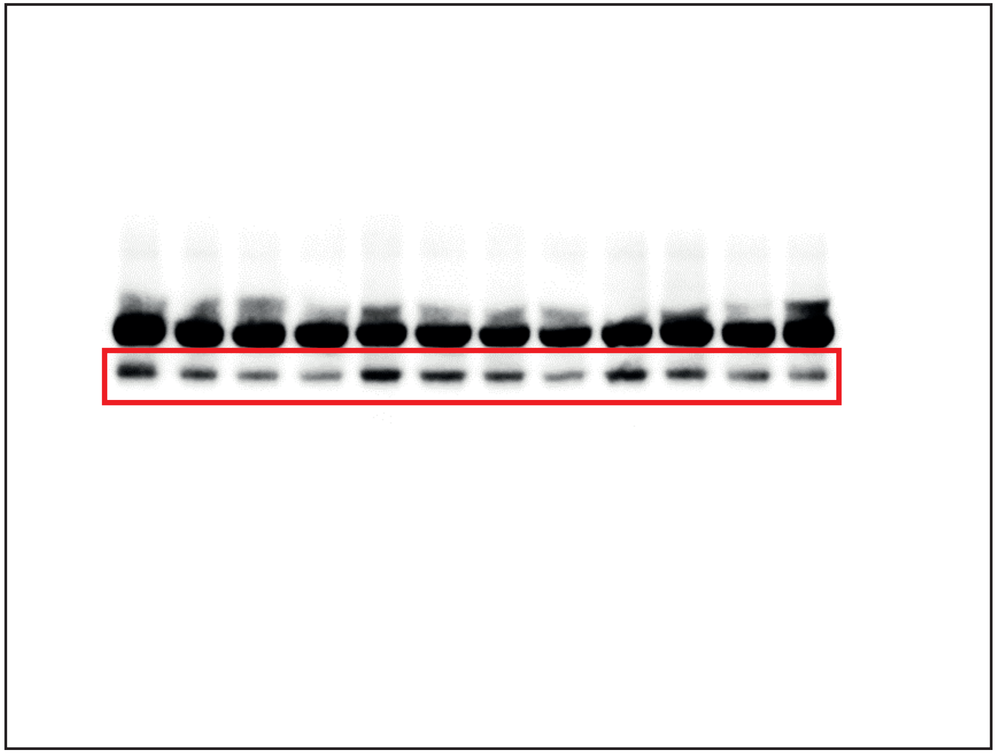

Full unedited gel of Fig3A, MTCO1

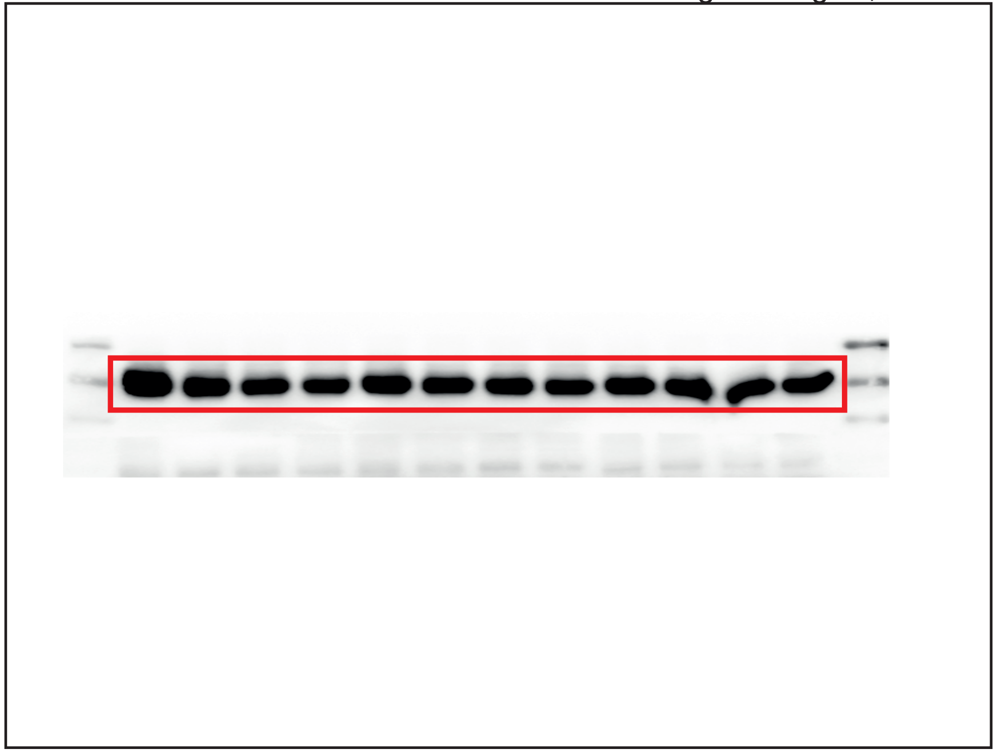

Full unedited gel of Fig3A, TUBULIN
