# Supplementary material for: Tetracycline-induced mitohormesis mediates disease tolerance against influenza
Source: J Clin Invest. 2022 Sep 1;132(17):e151540. doi: 10.1172/JCI151540 (PMC9433105; doi:10.1172/JCI151540)
Supplement: Supplemental table 3 [file jci-132-151540-s011.pdf]

No.	Name	Activity in hsp-6::gfp strain	Synthesis Method or Source
7 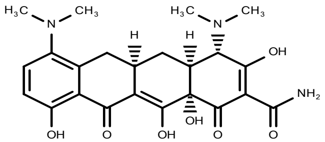	minocycline	inactive	Provided by Hovione, Sete 28asas 2674 – 506 Loures, Portugal
8 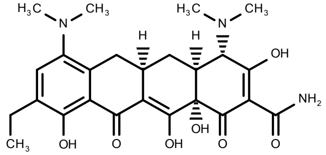	9-ethyl minocycline	inactive	**27**
9 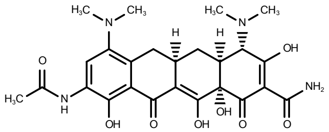	9-acetyl aminominocycline	inactive	**29**
10 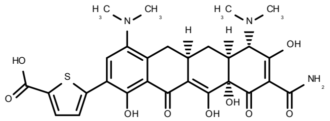	9-(4’-carboxy-1’-thiophene) minocycline	inactive	**27**
11 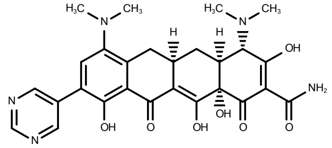	9-(5’-pyrimidyl) minocycline	inactive	**27**

No.		Name	Activity in hsp-6::gfp strain	Synthesis Method or Source
12	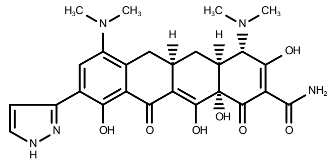	9-(3'-pyrazolyl) minocycline	inactive	**27**
13	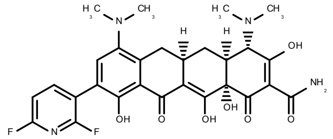	9-(3'-pyridinyl-2,6-difluoro) minocycline	inactive	**27**
14	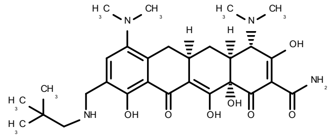	Omadacycline	inactive	**28**
15	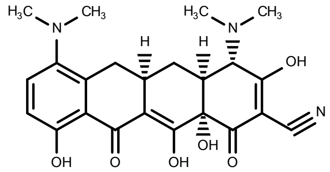	2N nitrile minocycline	inactive	**31**
16	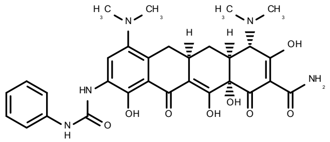	9-(phenylurea) aminominocycline	inactive	**35**

No.	Name	Activity in hsp-6::gfp strain	Synthesis Method or Source
17	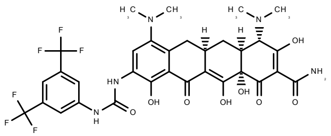 9-(3,5-trifluoromethylphenylurea) aminominocycline	inactive	**35**
18	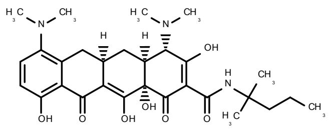 2N-1,1’-dimethylbutane minocycline	slight	**31**
19	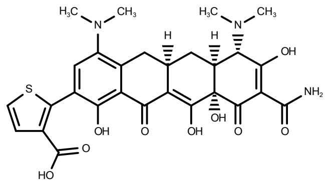 9-(2’carboxy-1’-thiophene) minocycline	inactive	**27**
20	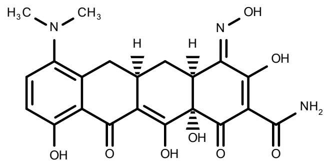 4-oxime minocycline	inactive	**35**
21	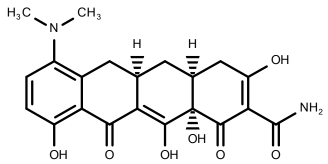 4-dedimethylamino minocycline	inactive	**27**

No.		Name	Activity in hsp-6::gfp strain	Synthesis Method or Source
22	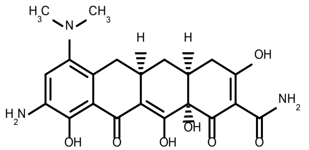	4-dedimethylamino-9-aminominocycline	inactive	**27, 29**
23	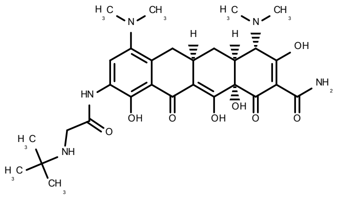	Tygacil™	slight	**29**
24	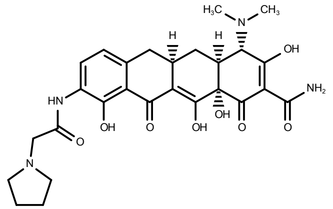	9-(pyrrolidinylmethylcarbonyl) aminominocycline	inactive	**29**
25	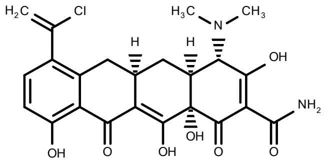	7-(1-chlorostyryl) sancycline	inactive	**27**
26	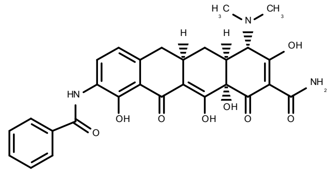	9-phenacyl aminosancycline	inactive	**29**

No.	Name	Activity in hsp-6::gfp strain	Synthesis Method or Source
27	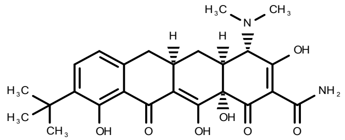 9-tert-butyl sancycline	inactive	**32**
28	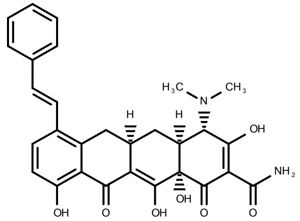 7-(1-styrylphenyl) sancycline	inactive	**27**
29	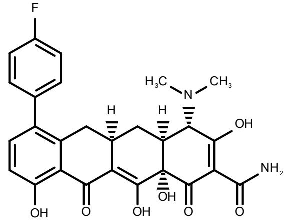 7-(4-fluorophenyl) sancycline	inactive	**27**
30	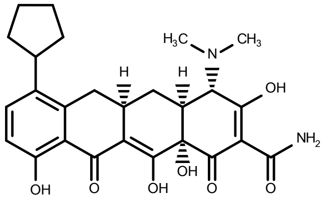 7-cyclopentyl sancycline	inactive	**27**

No.		Name	Activity in hsp-6::gfp strain	Synthesis Method or Source
31	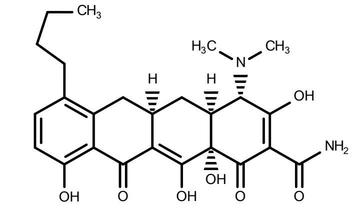	7-butyl sancycline	slight	**27**
32	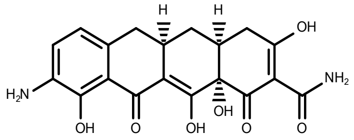	4-dedimethylamino-9-amino sancycline	inactive	**27**
33	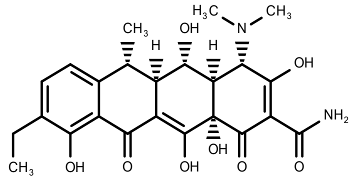	9-ethyl doxycycline	inactive	**27**
34	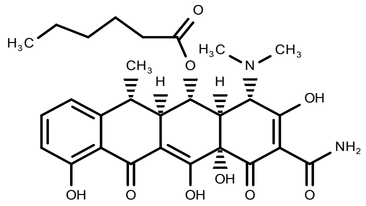	5-hexanoate doxycycline	slight	**32**
35	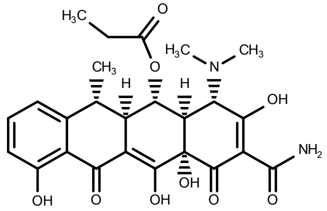	5-propionate doxycycline	inactive	**32**

No.	Name		Activity in hsp-6::gfp strain	Synthesis Method or Source
36	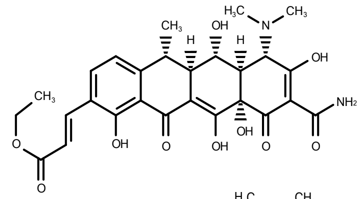	9-ethylacrylate doxycycline	slight	**27**
37	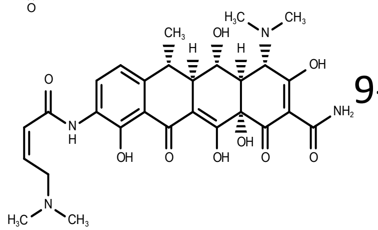	9-(3'-dimethylaminoacrylate) amino doxycycline	inactive	**29**
38	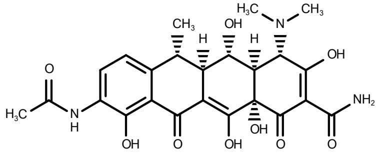	9-acetylamino doxycycline	inactive	**29**
39	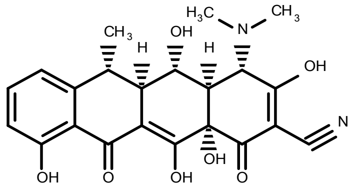	2N-nitrile doxycycline	inactive	**31**
40	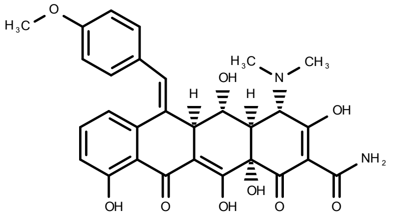	13-(4'-methoxyphenyl) methacycline	slight	**27**

No.		Name	Activity in hsp-6::gfp strain	Synthesis Method or Source
41	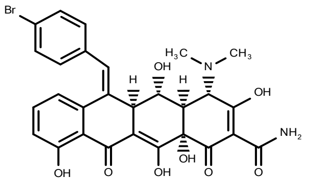	13-(4'bromophenyl) methacycline	slight	**27**
42	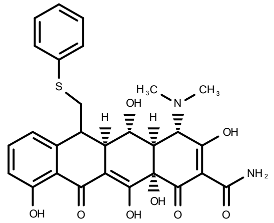	13-thiophenyl dihydro methacycline	slight	**26**
43	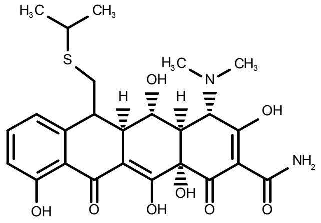	13-isopropylthio dihydro methacycline	slight	**26**
44	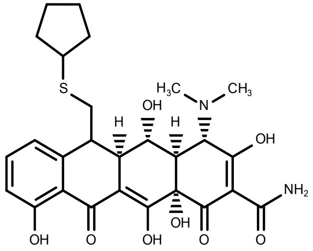	13-cyclopentylthio dihydro methacycline	slight	**26**
45	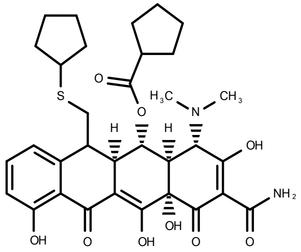	5-cyclopentanoate-13-cyclopentylthio dihydro methacycline	slight	**26, 32**

No.		Name	Activity in hsp-6::gfp strain	Synthesis Method or Source
46	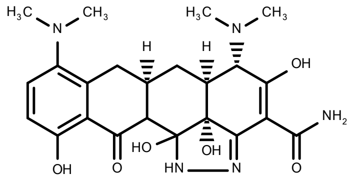	1,12- hydroxypyrazolo minocycline	inactive	**65**
47	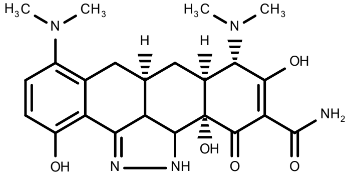	11,12-pyrazolo minocycline	inactive	**34**
48	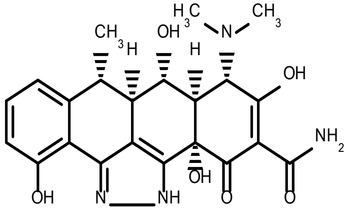	11,12-pyrazolo doxycycline	inactive	**34**
49	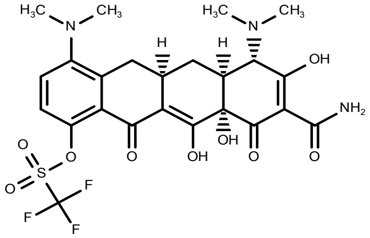	10-triflate minocycline	inactive	**Unpublished results**
50	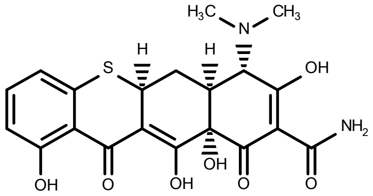	Thiatetracycline	inactive	**Gift from Pfizer, Inc. Groton, CT USA**

No.		Name	Activity in hsp-6::gfp strain	Synthesis Method or Source
51	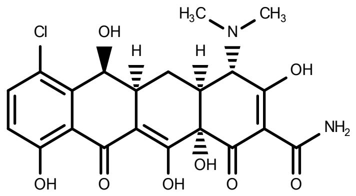	demeclocycline	slight	**Gift from Pfizer, Inc. Groton, CT USA**
52	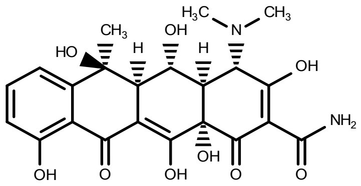	oxytetracycline	slight	**Gift from Pfizer, Inc. Groton, CT USA**

Compounds 1-39, 41-59, and 51-52 were prepared as the HCl salt form.
