# Supplementary material for: Tetracycline-induced mitohormesis mediates disease tolerance against influenza
Source: J Clin Invest. 2022 Sep 1;132(17):e151540. doi: 10.1172/JCI151540 (PMC9433105; doi:10.1172/JCI151540)
Supplement: Supplemental table 7 [file jci-132-151540-s015.pdf]

Table_S7

domain	mean	sd	min	max
**Bacteria**	99.923%	0.058%	99.687%	99.985%
**Eukaryota**	0.057%	0.042%	0.003%	0.199%
**Viruses**	0.02%	0.045%	<0.001%	0.299%
**Archaea**	<0.001%	<0.001%	<0.001%	<0.001%
