# Supplementary material for: Tetracycline-induced mitohormesis mediates disease tolerance against influenza
Source: J Clin Invest. 2022 Sep 1;132(17):e151540. doi: 10.1172/JCI151540 (PMC9433105; doi:10.1172/JCI151540)
Supplement: Supplemental table 8 [file jci-132-151540-s016.pdf]

Table_S8

TimePoint	Comparison	R2	p	p.adj	sign.sym.
**Day -4**	'veh' vs '9TB 1mpkd' vs 'Dox 40mpkd'	0.2542	0.1978	NA	ns
**Day 0**	'veh' vs '9TB 1mpkd' vs 'Dox 40mpkd'	0.5166	0.0081	NA	**
**Day 3**	'veh' vs '9TB 1mpkd' vs 'Dox 40mpkd'	0.4632	0.0032	NA	**
**Day -4**	'veh' vs '9TB 1mpkd'	0.2471	0.1451	0.2601	ns
**Day -4**	'veh' vs 'Dox 40mpkd'	0.0814	0.6908	0.6908	ns
**Day -4**	'9TB 1mpkd' vs 'Dox 40mpkd'	0.2791	0.1734	0.2601	ns
**Day 0**	'veh' vs '9TB 1mpkd'	0.1693	0.2821	0.2821	ns
**Day 0**	'veh' vs 'Dox 40mpkd'	0.6212	0.0293	0.0441	**
**Day 0**	'9TB 1mpkd' vs 'Dox 40mpkd'	0.4384	0.0294	0.0441	**
**Day 3**	'veh' vs '9TB 1mpkd'	0.245	0.1137	0.1137	ns
**Day 3**	'veh' vs 'Dox 40mpkd'	0.558	0.029	0.0847	*
**Day 3**	'9TB 1mpkd' vs 'Dox 40mpkd'	0.3119	0.0565	0.0847	*
